# Three Thousand Years of Continuity in the Maternal Lineages of Ancient Sheep (*Ovis aries*) in Estonia

**DOI:** 10.1371/journal.pone.0163676

**Published:** 2016-10-12

**Authors:** Eve Rannamäe, Lembi Lõugas, Camilla F. Speller, Heiki Valk, Liina Maldre, Jarosław Wilczyński, Aleksandr Mikhailov, Urmas Saarma

**Affiliations:** 1 Department of Archaeology, Institute of History and Archaeology, University of Tartu, Tartu, Estonia; 2 Archaeological Research Collection, Tallinn University, Tallinn, Estonia; 3 Department of Archaeology, University of York, York, United Kingdom; 4 Institute of Systematics and Evolution of Animals, Polish Academy of Sciences, Kraków, Poland; 5 Archaeological Center of Pskov Region, Pskov, Russia; 6 Department of Zoology, Institute of Ecology and Earth Sciences, University of Tartu, Tartu, Estonia; CSIRO, AUSTRALIA

## Abstract

Although sheep (*Ovis aries*) have been one of the most exploited domestic animals in Estonia since the Late Bronze Age, relatively little is known about their genetic history. Here, we explore temporal changes in Estonian sheep populations and their mitochondrial genetic diversity over the last 3000 years. We target a 558 base pair fragment of the mitochondrial hypervariable region in 115 ancient sheep from 71 sites in Estonia (*c*. 1200 BC–AD 1900s), 19 ancient samples from Latvia, Russia, Poland and Greece (6800 BC–AD 1700), as well as 44 samples of modern Kihnu native sheep breed. Our analyses revealed: (1) 49 mitochondrial haplotypes, associated with sheep haplogroups A and B; (2) high haplotype diversity in Estonian ancient sheep; (3) continuity in mtDNA haplotypes through time; (4) possible population expansion during the first centuries of the Middle Ages (associated with the establishment of the new power regime related to 13^th^ century crusades); (5) significant difference in genetic diversity between ancient populations and modern native sheep, in agreement with the beginning of large-scale breeding in the 19^th^ century and population decline in local sheep. Overall, our results suggest that in spite of the observed fluctuations in ancient sheep populations, and changes in the natural and historical conditions, the utilisation of local sheep has been constant in the territory of Estonia, displaying matrilineal continuity from the Middle Bronze Age through the Modern Period, and into modern native sheep.

## Introduction

### Background

Sheep (*Ovis aries*) were domesticated around 11 000 years ago in the area of the Fertile Crescent [1]. It has been suggested that sheep were initially reared for meat and milk (so-called ‘primitive’ sheep populations [2,3]) and only later for wool [2]. It has been demonstrated that early movements of domesticated sheep out of the Middle East and into Europe included predominantly ‘primitive populations’ while more recent movements included sheep with markedly improved wool production [3]. The latter were dispersed in several migratory episodes across Eurasia and Africa, where they gradually replaced majority of primitive sheep populations and provided the foundation for many modern breeds [3].

**Fig 1:**
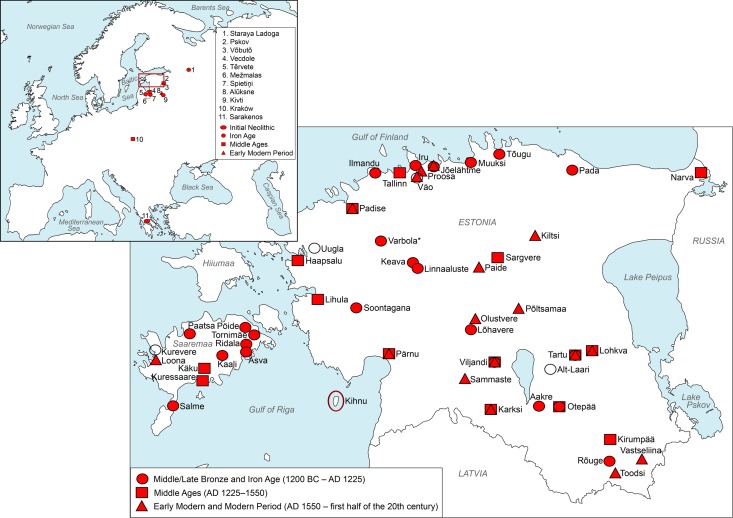
Geographic origin of ancient sheep samples analysed in this study. Samples (*n* = 134) come from Estonia (*n* = 115), Latvia (*n* = 7), Poland (*n* = 3), Russia (*n* = 7), and Greece (*n* = 2). The location of Kihnu Island in the Gulf of Riga for which the native sheep breed is named, is circled; the samples of Kihnu sheep (*n* = 44) were collected from the mainland population in south-western Estonia. Site marks on map can indicate several samples from one site; colourless marks indicate sites without any successful samples (for details see S1 Table).

As the wild ancestors of domestic sheep are absent in northern Europe, the first zooarchaeological evidence for sheep is thought to be associated with the introduction of domestic animals. In Estonia, the first zooarchaeological evidence for domestic sheep is a bone awl of sheep metacarpal bone recovered from a Late Neolithic Corded Ware (2900–1800 BC) burial, dated by associated finds to the beginning of that period, 2900–2700 BC [4]. This period likely marks the first steps of animal husbandry in the territory of present Estonia, although evidence for livestock husbandry remains sparse even into the Early (1800–1200 BC) and Middle (1200–850 BC) Bronze Age period. Nevertheless, by the Late Bronze Age (850–500 BC) animal husbandry had developed substantially, evidenced by abundant remains of cattle, sheep, goat, horse and pig in the archaeological record of settlement sites like Asva on Saaremaa Island [5] (Fig 1). The subsequent Early Iron Age (500 BC–AD 550) yields little osteological evidence for animal husbandry, which could be attributed both to the mixed nature of archaeological deposits and the dispersed settlement pattern characteristic of this period (making it difficult to locate sites on landscape). From the Late Iron Age (AD 550–1225) onwards, animal remains, especially of domesticates, again become numerous and are hereafter abundant in the archaeological deposits.

The time of the crusades in Estonia (AD 1208–1227) which marks the transition from the prehistoric to the historic era and the beginning of the Middle Ages (AD 1225–1550), brought significant changes in power structures and settlement. These decades are illustrated by the Chronicle of Henry of Livonia, where he describes ‘countless’ numbers of sheep or ‘other livestock’ besides cattle, oxen and horses, that were taken from the local inhabitants during the campaigns [6]. Medieval zooarchaeological material, however, comes predominantly from urban deposits; rural assemblages tend to be more rarely excavated and/or poorly preserved. Urban assemblages are dominated by domestic mammals like cattle, sheep, goat, and pig, while the exploitation of wild game is practically non-existent, in comparison to prehistoric times, *e*.*g*. [7]. Widening trade (*e*.*g*. through the German Hanse), the development of towns, and growing human populations all influenced animal husbandry, supposedly increasing the amount of livestock. The local livestock populations may have been augmented by imported individuals, although this practice would have been rather exceptional and occurred only in wealthy manors as known from the later periods.

The Middle Ages came to an end with the Livonian war (AD 1558–1583). Well dated zooarchaeological material from the 17^th^ and 18^th^ centuries is scanty, firstly, because of limited archaeological interest in the Early Modern Period (AD 1550–1800) the faunal remains are often not collected during the fieldwork, and secondly, it is difficult to archaeologically distinguish the layers of this period from more recent ones. The same issues apply to the following Modern Period (AD 1800 –first half of the 20^th^ century) which also marks the beginning of large-scale breeding. The wide use of Merino sheep after the Middle Ages is believed to have been the most influential factor in the development of modern breeds [8]. In Italy, Spanish Merino rams were used to improve local sheep as early as in 1435 [9], although in northern part of Europe it started much later: in Sweden from 1723 [2] and in Finland the import of ‘Spanish’ sheep with finer wool has been recorded from the 16^th^ century [10]. In Estonia, the first documents of foreign ‘English’ sheep breeds are known from the 1670s; around the same time some imported breeds have also been recorded in Latvia [11]. The first written documents referring to local Estonian sheep appear at the end of the 18^th^ century (1794), where small sheep with coarse wool, long slender legs and relatively short tails have been described [12]. At the same time, first attempts were made to improve local sheep with Spanish Merinos [12]. In 1824, the true breeding of fine-wool sheep in Estonia began in wealthier manors, with extensive improvement of local sheep first with Merino and then with other breeds like Shropshire and Cheviot; the latter two were used to develop the modern-day Estonian breeds, namely the Estonian Blackhead and Estonian Whitehead, respectively [12–15]. Among peasants, breed improvement started later, in the second half of the 19^th^ or in the beginning of the 20^th^ century, and at a more limited scale [12].

**Fig 2:**
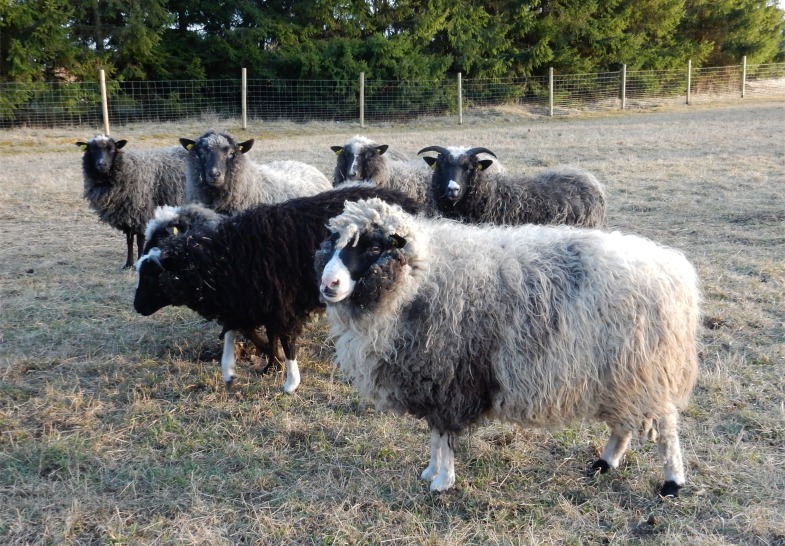
Kihnu native sheep. Note the different colours of the coarse wool (white, grey, black), slender un-woolly legs, and horns on both males (in front, on left) and females (behind, on right). Both males and females can also be polled or scarred (the light grey ram in front has scarred horns). Photo: Eve Rannamäe (April 2016).

‘Native’ sheep survived in peripheral areas of Estonia in spite the introgression of improved and imported breeds, and the neglect of local aboriginal sheep under more recent Soviet influence (*i*.*e*., during the period of collective and state farming in 1950s–1991). These sheep are now being actively revived, based predominantly on a relict population from Kihnu Island in the Riga Bay (Fig 1). The ‘Kihnu native sheep’ breed (Fig 2) was finally accepted as a licensed breed in January 2016 (Veterinary and Food Board, Estonia). Based on microsatellite analysis, Kihnu sheep have been shown to be genetically distinct from modern breeds and also from other primitive northern European native breeds (Tapio *et al*., in prep.), and ancient mitochondrial DNA (mtDNA) analyses have suggested a link between Kihnu and archaeological sheep populations in Estonia [16]. Similar circumstances of indigenous populations surviving on the peripheries have occurred in other parts of Europe, *e*.*g*. [17] and have been suggested to potentially represent descendants of the first migratory waves of primitive populations out of the Fertile Crescent [3].

### Aims of the Study

Analyses of archaeological sheep remains in Estonia have primarily relied on morphological methods, providing information about overall size and body type, as well as the consumption and utilisation of sheep and their by-products, *e*.*g*. [7,18–24]. Additionally, studies of archaeological textiles have revealed information about the local wool, which was two-layered, semi-coarse and variable in fibre coarseness, *e*.*g*. [25]. However, uneven representation of osteological material from different time periods, insufficient dating, and only partial analysis of many bone assemblages has made it difficult to draw wider conclusions about the development of sheep populations. Only in recent years molecular methods have been applied to study the genetic diversity of maternal lineages among Estonian ancient sheep. Mitochondrial DNA analysis of 31 ancient sheep remains dated *c*. 800 BC to AD 1700 demonstrated the presence of haplogroups A and B from the Late Bronze Age onwards, revealed several unique haplotypes and suggested higher mtDNA haplotype diversity during the Middle Ages as compared to the preceding and following periods [16].

Here, we build on previous ancient DNA (aDNA) studies by increasing both the targeted sequence length, and the number of samples from different archaeological contexts to more fully investigate the temporal fluctuations of sheep populations in Estonia, and explore the degree of continuity in maternal lineages from the Middle Bronze Age to recent breeding in the Modern Period.

Our first aim is to investigate the hypothesis of a shift in mitochondrial genetic diversity during the transition from the prehistoric to the historic period, *i*.*e*., from the Late Iron Age to the Middle Ages. This hypothesis is based in part on the preliminary ancient mtDNA results published by [16], but also on osteological evidence for an increase in sheep consumption, and a decrease in withers height in domestic livestock during the Middle Ages [7]. Our second aim is to establish the degree of continuity between present-day populations of Kihnu sheep and local populations dating back to the Bronze Age. For example, are there any chronological changes in the maternal genetic diversity in archaeological sites where sheep husbandry can be traced through several centuries? To this end, we analysed mtDNA from 115 archaeological sheep remains (including 28 samples first reported in [16] for which we sequenced additional fragments of mtDNA) recovered from 71 archaeological sites in Estonia and dated from around 1200 BC to AD 1900s (Fig 1; S1 Table).

To provide comparative ancient material from neighbouring northern, eastern and southern areas, and from the area closer to the domestication centre, we also extracted mtDNA from 19 ancient sheep from 11 archaeological sites in Latvia, Russia, Poland, and Greece, dated from around 6800 BC to AD 1700 (Fig 1; S1 Table). We complemented this dataset with previously published sequences from Finland to assess the affinities of maternal sheep lineages between Estonia and Finland through time.

Our results provide additional basis for subsequent studies on maternal genetic variation in sheep and contribute to the understanding of the history of sheep husbandry not only in Estonia but also in broader areas in the north-eastern part of Europe.

## Materials and Methods

### Ancient Samples and Archaeological Sites

Samples (*n* = 134) of archaeological sheep bones were obtained from archaeological collections in Estonia (71 sites; *n* = 115), Latvia (6 sites; *n* = 7), Russia (3 sites; *n* = 7), Poland (1 site; *n* = 3), and Greece (1 site; *n* = 2) (Fig 1; S1 Table). Sampling permission was obtained from each institution housing the zooarchaeological collections (listed in S1 Table).

Estonian samples come from both urban and rural contexts–towns, castles, manors, monasteries, hillforts, settlement sites, field remains and burials–dating from the Middle Bronze Age to the 20^th^ century. The majority of the samples come from the mainland with fewer from the Saaremaa Island. Micro-regions yielding sheep remains over a long chronological span were preferentially targeted to identify temporal changes within a site or region, including Viljandi-Karksi, Tartu-Lohkva and Saaremaa Island. All samples were dated based on archaeological context or radiocarbon dating (S1 Table and S2 Text). Although sample selection aimed for homogeneous geographical and temporal coverage, it was constrained by standard archaeological contingencies of preservation and accessibility, plus the limitations of taxonomical identification of sheep remains.

Regions outside Estonia were not systematically sampled, but included primarily as comparative data. Most samples were contemporaneous with the Estonian data-set: Polish and Russian samples come from medieval towns Kraków, Pskov and Staraya Ladoga, of which the latter is concurrent with the Estonian Late Iron Age; Latvian samples come from settlement sites, hillforts, and castles–and represent time periods from the Iron Age to Early Modern Period. Initial Neolithic samples from Sarakenos cave in Greece precede the Estonian oldest samples by five thousand years, making them the oldest in the dataset (S1 Table).

Because of the abundance of bones in the faunal assemblages compared to teeth, as well as the increased ability to distinguish sheep from goats based on bone elements, most samples were bones, with only one tooth selected. For samples from the same archaeological deposit it was confirmed that each individual was sampled only once, by (a) morphological characteristics such as overall size and degree of epiphyseal fusion of long bones (used to estimate age at death of the animal [26]), and (b) single nuclear polymorphisms (SNPs) in mtDNA sequences.

All Estonian, Latvian, Russian, Polish, and Greek specimens are reported here for the first time, except 28 Estonian samples that were already included in a pilot study by [16], where a 213 base pair (bp) long fragment of the mitochondrial D-loop was targeted (fragments 1–2); in the current study we extended these sequences (fragments 3–5; S1 and S2 Tables).

### Ancient DNA Extraction

Sample preparation and DNA extraction of archaeological sheep bones were conducted in the dedicated aDNA laboratory at the University of Tartu. We followed the silica spin column protocol first described by [27] and amended by [10] with slight modifications (S1 Text). Briefly, 0.5 ml of bone powder was incubated overnight in an extraction buffer of 0.45M EDTA pH 8.0, 1M of Urea, and 200 μg of proteinase K at 55°C. The resulting supernatant was concentrated using Amicon Ultra-4 30kDa Centrifugal Filter Units (Merck Millipore, Darmstadt, Germany) and purified using QIAquick PCR Purification Kit (Qiagen, Hilden, Germany). A total of 150 μl of DNA solution was eluted from each sample for subsequent polymerase chain reaction (PCR) amplification.

A 599 bp fragment of mtDNA hypervariable D-loop control region corresponding to positions 15957–16556 in GenBank accession NC001941 [28], was amplified using five sheep-specific primer pairs yielding overlapping sequences [10] (S2 Table). PCR amplification was carried out with HotStarTaq Master Mix Kit (Qiagen, Hilden, Germany) in 25 μl reaction mix using 3 μl DNA extract, 0.2 μM of each primer, and 0.25 units (U) of Uracil DNA Glycosylase (Sigma-Aldrich). PCR conditions were: 37°C for 10 min; 95°C for 15 min; 55 three-step cycles of 94°C for 30 s, 58°C for 40 s and 72°C for 1 min; and final extension of 72°C for 10 min. Each DNA extract was amplified at least twice. All samples with successful PCR products were sequenced in both directions to achieve a minimum of two identical amplicons. PCR product purification and sequencing was performed following [29], and using the same primers as for the initial PCR.

### Authenticity of the Ancient DNA Results

Sample preparation and DNA extraction followed strict protocols for in-laboratory contamination control and detection [30–32]: (1) samples were prepared and extracted in a laboratory dedicated to aDNA; (2) pre-PCR and post-PCR analyses were conducted by different people in different laboratories located in separate buildings; (3) blank extracts and negative controls were incorporated into extractions and PCRs; (4) multiple haplotypes were observed, including within extraction batches that suggests a lack of cross-sample contamination; (5) repeated extractions of 28 bone samples were performed independently in two aDNA laboratories: at the University of Tartu, Estonia and at the Natural Resources Institute Finland, Finland using the same protocols and primer sets; repeated extractions of seven bone samples were performed at the University of Tartu, Estonia (S1 Table); all repeat PCR amplifications yielded the same sequences; (6) possible C→T and G→A substitutions [33–34] were avoided by using Uracil DNA Glycosylase in the PCR reaction; (7) only reproducible results were included in the analyses.

### Modern Samples of Kihnu Native Sheep

Previously collected blood samples of Kihnu sheep (*n* = 44) were kindly provided by veterinarian Anneli Ärmpalu-Idvand (Kihnu Native Sheep Society; no permits and ethical approval were required for the sampling). DNA was extracted in the University of Tartu using the High Pure PCR Template Preparation Kit (Roche Diagnostics, Mannheim, Germany) according to the manufacturer’s instructions. A 1451 bp fragment of mtDNA control region was amplified with one primer pair (S2 Table). PCR was performed in 20 μl containing 20–80 ng of DNA, 0.5 μM of each primer, 1x Advantage-2 PCR Buffer (BD Biosciences, Franklin Lakes, NJ, USA), 0.2 mm dNTP (Thermo Fisher Scientific, Waltham, US) and 0.5x Advantage-2 Polymerase Mix (BD Biosciences). Cycling parameters were: 95°C for 1 min, followed by 10 touch-down cycles at 95°C for 30 s, 60°C for 30 s (temperature was reduced by 0.5°C in each cycle), 68°C for 2 min, followed by 25 cycles: 95°C for 30 s, 55°C for 30 s and 68°C for 2 min. The final extension was 5 min at 68°C. PCR product purification and sequencing procedures followed [35]. Sequencing was carried out for both DNA strands using the same primers as for the initial PCR.

### Sequence Analysis

The obtained ancient sequences were edited using Geneious v.6.1.7. ([36], http://www.geneious.com). Multiple alignments of the ancient and modern sequences were conducted using ClustalW (cost matrix IUB, gap open cost 10, gap extend cost 0.2) through Geneious. After the removal of primer sequences, the alignment was truncated to 559 bp (region 15978–16536 in GenBank accession NC001941).

The number of haplotypes (*h*), haplotype (*Hd*) and nucleotide diversities (*π*), and Tajima’s D values (*D*) for each cohort were calculated with DnaSP v.5.10 [37]. Median-joining networks of the obtained D-loop haplotypes were produced through Network v.4.6.1.3. ([38], www.fluxus-engineering.com) with default values.

We assessed the continuity of haplotypes through time using a three-dimensional statistical parsimony network through TempNet [39] implemented through the R package v.3.1.2 [40]. We calculated the phylogenetic relationship between temporal cohorts, pairwise population fixation index (*F*_*ST*_) values [41] and analysis of molecular variation (AMOVA) using Arlequin v.3.5.1.2 [42].

The analysis was approached from two perspectives. Our primary focus was on Estonian material in order to assess the presence, continuity, and changes of unique and shared haplotypes through time. This was assessed by calculating the genetic diversity values and assessing the haplotype continuity for four temporal cohorts (*n* = 86; alignment length 559 bp): (1) Middle/Late Bronze and Iron Age (hereafter Bronze/Iron Age; *n* = 28), (2) Middle Ages (*n* = 39), (3) Early Modern and Modern Period (*n* = 19), and (4) Kihnu sheep (*n* = 44). Note that two samples that could not be assigned to either the Iron Age or Middle Ages (51OaVar1 and 67OaOte3; S1 Table), were omitted from temporal analysis. To further study the haplotype continuity within particular archaeological region, samples from Viljandi-Karksi (*n* = 21), Tartu-Lohkva (*n* = 12), and Saaremaa Island (*n* = 10) were analysed separately by a three-dimensional statistical parsimony network.

The secondary focus of the analysis was the comparison of Estonian ancient and modern data with other regions in Europe. A median-joining network was calculated with the Latvian, Russian, Polish and Greek ancient samples of this study (*n* = 14), and available sequences from GenBank–Finnish ancient (*n* = 26) and modern (*n* = 32) sheep sequences by [10]. Although the study sought to compare the Estonian data to previously published ancient sequences from neighbouring regions, *e*.*g*. [10,43–46], only those of [10] (samples from AD 800–1800) targeted homologous regions to our study. This final dataset was made up of 204 sequences trimmed to 523 bp.

## Results and Discussion

### PCR Amplification and DNA Preservation of Ancient Samples

In total, PCR amplification of all five primer sets was successful for 102 of the 134 sampled archaeological sheep specimens dating from *c*. 6800 BC to AD 1900s (76% success rate). Amplification failed completely for 22 samples, including two specimens reported in [16]; and was only partially successful (418–509 bp) for 10 samples–these were omitted from further analyses. All full and partial sequences were submitted to GenBank (accession nos. KP052793–KP052807 and KP052809–KP052815 for the updated 22 sequences reported first in [16]; KU670230–KU670319 for the 90 sequences newly reported in this study; and KX056139–KX056146 for the eight haplotypes of the 44 Kihnu sheep sequences; S1 Table).

No significant differences in the preservation of DNA were detected in temporal or spatial cohorts (S1 and S3 Tables). All successfully amplified ancient samples were consistent with morphological species identification of *Ovis aries*; no other species (*e*.*g*. domestic goat, *Capra hircus*) were identified. In general, the relatively high success rate for DNA amplification can be attributed to the recent antiquity of the samples, the correct osteological identification of species, as well as the taphonomic and climate conditions conducive to DNA preservation in Estonian ground.

### European Network

Surveys of ovine mtDNA variability have supported a broad genetic base during domestication [8]. Variation of hypervariable D-loop region of the mtDNA has defined at least five lineages (A–E) within modern breeds. Of these, haplogroups A and B are with wide global dispersal and located in every studied region, first documented by [47]. Haplogroup C is more common in Asia, the Fertile Crescent, Caucasus and Iberian Peninsula, *e*.*g*. [48–50]. Haplogroups D and E are the most recent discoveries and restricted to Middle East, Caucasus and Turkey, *e*.*g*. [49,51–52]. All five haplogroups have also been detected in ancient populations [10,16,43–45,53–58].

Previous studies on modern sheep genetics have shown that genetic diversity indices of the first domestic populations in the initial region of domestication have been very high and decreased with geographic distance from the centre [59–61]. This association is not always strong, which may be due to the extensive introgression of Merinos after the Middle Ages, resulting in extensive haplotype sharing of the modern breeds [8]. However, ancient DNA studies in the north-eastern Baltic Sea region have confirmed the tendency for decreasing genetic diversity with increasing physical distance from the domestication centre for cattle [62] and for sheep [16]. A recent study by [49] has also suggested strong historical human-mediated gene flow between breeds across eastern Eurasia, and proposed that sheep have spread with two different migratory waves: lineages A and B at *c*. 6400–6800 years ago, and lineage C at *c*. 4500 years ago.

**Fig 3:**
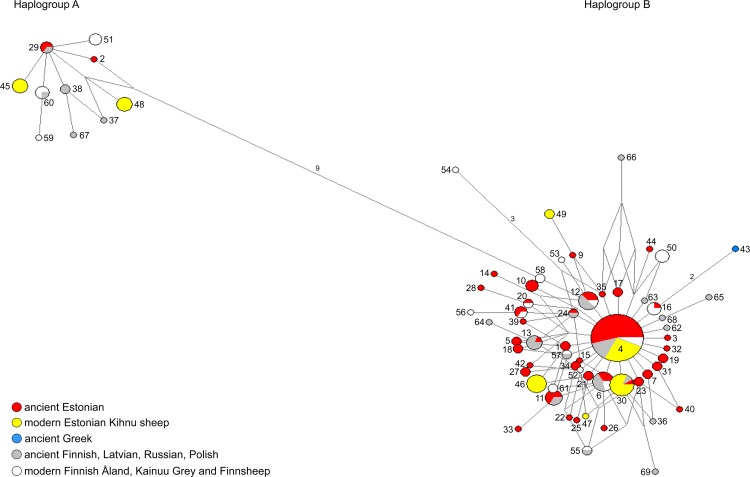
The median-joining network of 523 bp mtDNA D-loop haplotypes depicting the relationships between Estonian, Latvian, Russian, Polish, Greek, and Finnish ancient and modern sheep. The samples in the network are: Estonian (*n* = 88), Latvian (*n* = 5), Russian (*n* = 6), Polish (*n* = 2), and Greek (*n* = 1) ancient, and Estonian modern Kihnu sheep (*n* = 44) of our study; and Finnish ancient (*n* = 26) and modern (*n* = 32) samples from [10]. The numbers of the haplotypes are according to S6 Table. The size of the given node is proportional to the number of samples represented in a haplotype, with the smallest node representing a single individual. Branch length is proportional to the mutational distance; only mutational distances greater than 1 are indicated. For an elaborated time-period specific network see S1 Fig.

Latvian, Russian, Polish and Greek specimens were incorporated into the present study to provide a wider context for ancient sheep in Europe, in addition to ancient and modern Finnish samples from GenBank [10]. In total, 49 *Ovis aries* mtDNA haplotypes were defined by 40 variable sites (S5 Table) within the complete ancient and modern dataset. Following the standard classification of the sheep mtDNA haplogroups [52], 15 sequences were assigned to haplogroup A (10%) and 131 to haplogroup B (90%). In general, a high level of maternal diversity was observed, with 51% of the haplotypes being found in only one individual. A median-joining network of this larger dataset clearly cluster the sequences into these two haplogroups, where the central haplotype *H4* (Fig 3; S1 Fig; S6 Table) appears to be the founder of the lineage, from which the other haplotypes radiate with relatively few mutation steps. Some region-specific haplotypes become apparent, like (a) haplotypes represented by Kihnu sheep, (b) haplotype associated with the oldest sample in the dataset–the Greek sheep, and (c) haplotypes containing modern Finnish sheep breeds.

The Sarakenos sheep from the Initial Neolithic Greece (123OaSara2), which age was determined on the basis of a series of radiocarbon dates of charcoal to *c*. 6800 BC, is one of the earliest known sheep specimens in that region [2,63–64], and represents a very early stage of domesticated sheep dispersal into Europe. The Sarakenos cave is a site where sheep and goats seem to have appeared suddenly and have immediately dominated the faunal assemblage, being clearly a food waste, possibly by Neolithic shepherds [65]. In our network the Sarakenos sheep has a unique haplotype (*H43*) which is one of the most distant from the central haplotype in haplogroup B.

Comparisons between Estonian and Finnish sheep reveal 10 shared haplotypes, while 20 haplotypes are specific to Finland and 33 to Estonia (Fig 3; S1 Fig; S6 Table). In haplogroup A one post-medieval (= early modern) Finnish sample is shared with two Estonian Iron Age samples. In haplogroup B, in addition to the central haplotype, ancient Estonian from all time periods are shared with ancient Finnish sequences, as well as with modern Finnsheep. A large proportion of Kihnu sheep belong to the most abundant haplotype *H4*, sharing common ancestry with sheep from broad geographical and temporal distribution. Kihnu sheep have common ancestry also with medieval Russian and the Iron Age Estonian sheep (*H30*). However, there are also several haplotypes which are unique to Kihnu sheep (*H45*–*H49*). Interestingly, none of the Estonian samples share haplotypes with other modern Finnish breeds such as Kainuu Grey and Åland sheep. Likewise, none of the Finnish sheep (either ancient or modern), share haplotypes with the Estonian Kihnu sheep (except in the central haplotype of haplogroup B). In summary, Estonian ancient sheep show an affinity to eastern, southern, and northern European ancient sheep, as well as to native Finnsheep, while the Kihnu, Kainuu Grey and Åland native breeds appear to be genetically more distinct.

### Mitochondrial Diversity and Continuity in Estonian Sheep

**Fig 4:**
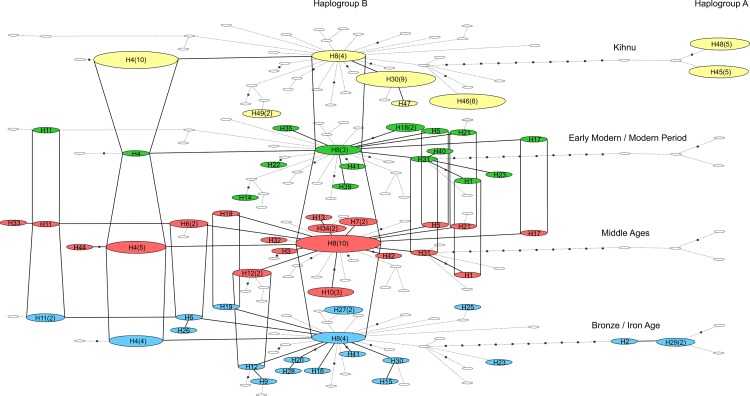
Three-dimensional network of Estonian ancient and modern Kihnu native sheep from four time periods. A three-dimensional statistical parsimony network was calculated using TempNet based on 559 bp mitochondrial D-loop haplotypes of Estonian sheep (total *n* = 86, *h* = 45) dating to the Bronze/Iron Age (blue), Middle Ages (red), and Early Modern / Modern Period (green), as well as Kihnu sheep (*n* = 44, *h* = 8; yellow). The size of the given node is proportional to the number of samples represented in a haplotype, with the smallest node representing a single individual; number of samples greater than one is indicated in the parentheses. Branch length is not proportional to the mutational distance; mutational distances greater than 1 are indicated with black dots. Colourless nodes denote haplotypes absent within the time period.

Among the ancient and modern Estonian specimens, 45 mtDNA haplotypes were observed (47% unique haplotypes), again dominated by haplogroup B (*n* = 119, 90%), with a lower frequency of haplogroup A (*n* = 13, 10%). The three-dimensional network outlines the central structure of the haplotypes of Estonian sheep through time (Fig 4). Two of the haplotypes, *H4* and *H8*, are continuous through all four periods. Other haplotypes disappear or emerge in time, and some, due to sampling effect, are discontinuous between the periods. This extensive continuity of core mtDNA haplotypes may be result of husbandry practices favouring the maintenance of female animals, who were kept in large numbers for population reproduction and milk production. Comparison of the maternal lineages and the shared haplotypes from as early as the Middle Bronze Age strongly suggest the affinity between Kihnu sheep and haplotypes characteristic to ancient sheep.

**Fig 5:**
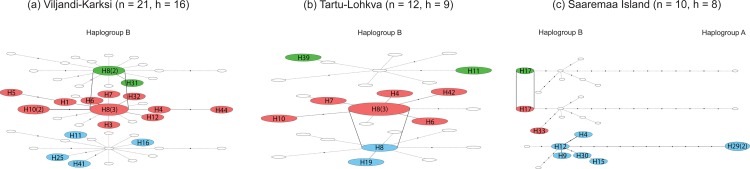
Three-dimensional network of Estonian ancient sheep from three micro-regions through three time periods. A three-dimensional statistical parsimony network was calculated using TempNet based on 559 bp mitochondrial D-loop haplotypes of Estonian sheep from three micro-regions: (a) Viljandi-Karksi, (b) Tartu-Lohkva and (c) Saaremaa Island dating to the Bronze/Iron Age (blue), Middle Ages (red), and Early Modern / Modern Period (green). The size of the given node is proportional to the number of samples represented in a haplotype, with the smallest node representing a single individual; number of samples greater than one is indicated in the parentheses. Branch length is not proportional to the mutational distance; mutational distances greater than 1 are indicated with black dots. Colourless nodes denote haplotypes absent within the time period.

None of the continuous lineages (*e*.*g*. *H4*, *H8*, *H11* in Fig 4) appear to be region-specific. Therefore, we attempted to test whether we would detect continuity in the selected micro-regions. Two Teutonic Order related sites–Viljandi (castle, town and suburb) and Karksi (castle)–formed a dual power center with close hinterlands during the Middle Ages [66] and were of special interest in this study, as zooarchaeological studies at these sites have shown changing consumption patterns, and a decrease in the wither’s height of the main livestock during the Middle Ages, *e*.*g*. [7,18–19,24]. The temporal haplotype network of Viljandi-Karksi region (Fig 5A) reveals that no prehistoric haplotypes continued into medieval period. Interestingly, the same pattern is seen for Saaremaa Island (Fig 5C), but not for Tartu-Lohkva micro-region (Fig 5B). However, since the sample sizes are small here, caution should be taken not to over interpret the results.

**Fig 6:**
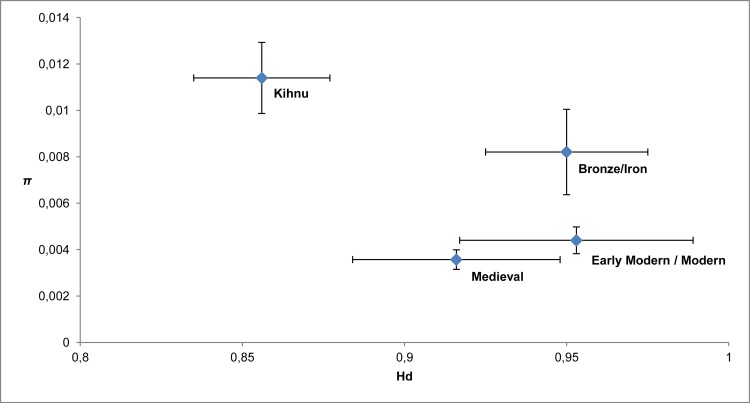
Scatterplot diagram for nucleotide (*π*) and haplotype (*Hd*) diversities of the Estonian ancient and modern Kihnu native sheep by time period. Calculations are based on 559 bp mtDNA D-loop sequences from four temporal cohorts of Bronze/Iron Age (*n* = 28), Middle Ages (*n* = 39), Early Modern / Modern Period (*n* = 19) and Kihnu sheep (*n* = 44). Error bars indicate standard deviation (Table 1).

We expected to observe significant fluctuations in the genetic diversity of ancient sheep mtDNA in Estonia from Bronze Age to Modern Period, as the animal husbandry would have followed the expansions and declines of livestock populations–for example due to human migrations, wars or ecological changes. In particular, our expectations were that medieval populations would display significantly higher mitochondrial diversity compared to other time cohorts, as suggested in [16]. The opposite pattern, however, was observed in our study. The medieval cohort displayed the lowest genetic diversity indices within the ancient cohorts, whereas the highest haplotype diversity was observed within the Early Modern / Modern Period. Contemporary Kihnu sheep are characterized by the lowest haplotype diversity and the highest nucleotide diversity. Overall, the comparison of haplotype and nucleotide diversity indices revealed no significant differences among the temporal cohorts (Fig 6; Table 1). The general homogeneity among time periods may reflect the hardiness and continuity of sheep populations through time. Although famines, plagues and murrains were often devastating to the populations of both human [67–68] and livestock, it has been argued that the recovery of livestock after these events was rather rapid [69]. Primitive and hardy sheep are usually more likely to survive harsh climatic conditions, including cold winters with only dry leaves for feed [70]. However, this pattern in genetic diversity among temporal populations may also reflect the difficulty in identifying short-term events in zooarchaeological remains due to a lack of resolution in the archaeological stratigraphy.

**Table 1:** Statistics for four temporal cohorts of Estonian ancient and modern Kihnu native sheep by time period.

Time cohort	*n*	*S*		*h*		*Hd*		*π* ± SD	*D*
		(gaps not con.)	(gaps con.)	(gaps not con.)	(gaps con.)	with standard deviation SD (gaps not con.)	(gaps con.)	with standard deviation (gaps not con.)	(gaps not con.)
**Bronze/Iron Age**	**28**	26	**27**	18	**19**	**0.950 ± 0.025**	0,96032	**0.00821 ± 0.00184**	**-1.13699**
**Middle Ages**	**39**	21	**21**	20	**20**	**0.916 ± 0.032**	0,91633	**0.00357 ± 0.00042**	-2.00057*
**Early Modern / Modern Period**	**19**	15	**16**	14	**16**	**0.953 ± 0.036**	0,97661	**0.00440 ± 0.00058**	**-1.59901**
**Kihnu**	**44**	21	**21**	8	**8**	**0.856 ± 0.021**	0,85624	**0.01140 ± 0.00153**	**1.03872**

The calculations are based on 559 bp mtDNA D-loop sequences. Results are presented according to whether gaps were not considered (*gaps not con*.) or considered as the fifth state (*gaps con*.) in the calculations. Number of samples (*n*), number of variable sites (*S*), number of haplotypes (*h*), haplotype diversity (*Hd*), nucleotide diversity with standard deviation (*π* ± SD), and Tajima’s D (*D*) are shown. Values used in the subsequent analyses and discussion are shown bold.

* Statistical significance at *p* < 0.05.

### Population Expansion in the Middle Ages

Although the medieval period is not characterised by increasing genetic diversity indices, interestingly, Tajima’s D values seem to suggest a population expansion during this period (*D* = –2.00057, *p* < 0.05; Table 1; [71]). To verify this result, we eliminated the possible bias caused by uneven length of the temporal cohorts, as Bronze/Iron Age (2225 years) yields samples from far longer period than the Middle Ages (325 years). We decreased the number of sequences only to the ones from the later centuries of the Late Iron Age (AD 900–1225), and also divided the medieval period into two–the earlier (AD 1225–1400) and the later (AD 1400–1550) part. We then compared the genetic diversities and Tajima’s D between those groups. The results confirmed a potential population expansion in the first period of the Middle Ages (*D* = –2.12072, *p* < 0.01), but showed no significant changes between the last centuries of the Late Iron Age and the first half of the Middle Ages (χ^2^ = 13.371, *p* = 0.4975 *df* = 14) or between the earlier and later part of the Middle Ages (χ^2^ = 12.504, *p* = 0.5659, *df* = 14).

These results correlate with the increasing utilisation of livestock seen in the medieval osteological data. Archaeological and zooarchaeological data suggest that as human population expanded, so did the demand for agricultural products, resulting in subsequent expansion of local livestock populations. The establishment of new power structures starting from the 13^th^ century created a degree of social polarisation between the colonists and the colonised, arguably with a stronger impact on the local society than the crusades [72–73]. Nevertheless, as much of the local population–the farmers (and their livestock)–remained intact, the economic basis remained consistent, with new power centres dependent on indigenous population and their farming [72–73].

Livestock expansion could be associated with the expanding trade as well, but assessing the degree of livestock trade during the Middle Ages is complicated, largely due to a lack of documentary evidence. During the Middle Ages sheep products, wool and textiles particularly, were very important in western Europe [2]. However, in Livonia there are no traces or documentation of medieval wool trade, instead the textiles were imported from western Europe, and in the rural areas local wool in the textile manufacture prevailed all through the Middle Ages [25]. Furthermore, land-routes between the Hanseatic towns in western Europe and Livonia were not favoured for trade, instead sea-routes were more common [74]–by which an extensive sheep trade seems unlikely (and not supported by written documentation). The occasional import of breeding rams, however, could be speculated to have occurred also in medieval Livonia, as known from other countries, *e*.*g*. [9–10], but these individuals would likely have had a limited effect on the local gene pool [8,10], and certainly would not be visible in the maternal lineages. Although colonizers may have brought in new livestock, in Livonia the subsequent development of sheep populations was most probably autonomic, especially in the first centuries after the conquest (see also [2]).

Overall, the mitochondrial genetic results obtained in this study support the historic and archaeological evidence for population expansion of local sheep, rather than significant introduction of imported animals during the medieval period.

### Modern Kihnu Native Sheep Compared to Ancient Populations

Our results suggest that following the Middle Ages, the indigenous sheep population in Estonia continued to expand until they became part of the large-scale breeding process in the 19^th^ and 20^th^ century. Due to decrease in the use of local sheep and mixing them with modern imported breeds, local sheep were driven to almost extinction. This population decline was a gradual process which reached its low point during the last decades of the 20^th^ century and is now reflected in the genetic diversity estimates of the Kihnu sheep analysed in this study. As revealed by the genetic differentiation estimates shown in Table 2 (and S4 Table), Kihnu sheep are significantly different than the ancient sheep cohorts, while no significant difference is observed amongst ancient cohorts. What might account for the genetic distinctiveness of the Kihnu sheep? First, the decrease in population size during the last (two) hundred years may be reflected in the relatively high value of Tajima’s D in Kihnu sheep (*D* = 1.03872, *p* > 0.10, not significant), as one possible interpretation for this high value could be recent population contraction [71]. Secondly, Kihnu sheep samples in this study are all descended from a population from the Kihnu Island, recently acknowledged as a native breed that is currently distributed across Estonia. This relatively small founding population, however, could have caused genetic drift, which has resulted in somewhat lower haplotype diversity (0.856 in Kihnu sheep) compared to the Early Modern / Modern cohort (0.953). However, Kihnu sheep have considerably higher nucleotide diversity (*π* = 0.01140) compared to the ancient cohorts. The latter coincides with the high number of novel haplotypes among Kihnu sheep and might reflect the accumulation of mutations during population development [54].

**Table 2:** Genetic differentiation estimates between four temporal cohorts of Estonian ancient and modern Kihnu native sheep.

	Bronze/Iron Age	Middle Ages	Early Modern / Modern Period
**Bronze/Iron Age (*n* = 28)**	-	-	-
**Middle Ages (*n* = 39)**	χ^2^ = 35.843 *p* = 0.2929 (*df* = 32)	-	-
**Early Modern / Modern Period (*n* = 19)**	χ^2^ = 29.640 *p* = 0.4322 (*df* = 29)	χ^2^ = 30.121 *p* = 0.3087 (*df* = 27)	-
**Kihnu (*n* = 44)**	χ^2^ = 47.775 *p* = 0.0018* (*df* = 23)	χ^2^ = 58.148 *p* = 0.0002** (*df* = 25)	χ^2^ = 50.545 *p* = 0.0003** (*df* = 21)

Calculations were made in DnaSP by Pearson’s chi-squared test (χ^2^). The statistical significance (*p*) and the degrees of freedom (*df*) are shown.

* Statistical significance at 0.001 < *p* < 0.01.

** Statistical significance at *p* < 0.001.

We recognize a potential bias in our grouping of temporal cohort, as Kihnu sheep represent a contemporaneous ‘living population’, while the ancient samples are drawn from a group of individuals living over several centuries, and thus may have artificially inflated diversity values. Also, the time periods used in the analyses are not of the same duration: 2225 years for Bronze/Iron Age, 325 years for medieval and 450 years for Early Modern / Modern Period. To test the possible bias for the longest, prehistoric cohort, five of the oldest samples from the Bronze and Early Iron Age were left out and statistical significances for genetic differences were re-calculated only with sequences from the Late Iron Age (*n* = 22; period length 700 years). Using this shortened time span, the Late Iron Age still displayed no significant differences with any of the populations except for the Kihnu sheep (χ^2^ = 43.851 *p* = 0.0024, *df* = 21).

Although we recognize that mtDNA genetic diversity indices are only one part of the genetic puzzle, this study provides the basis for future analyses based on Y-chromosome, or nuclear SNPs to assess the extent of genetic diversity loss within this valuable native breed.

## Conclusions

In this study, we sought to extract mtDNA from archaeological sheep remains and establish a baseline for genetic diversity in ancient sheep in Estonia. Our results point to excellent mtDNA preservation of osteological remains in various archaeological sites in Estonia, within mitochondrial lineages falling into the haplogroups (A and B) observed in other parts of north-eastern Europe.

Based on previous ancient DNA and osteological data, we expected to observe temporal fluctuation in sheep genetic diversity, particularly within the medieval period. Instead, we identified relative stability in genetic diversity and continuity in the ancient matrilines from Middle Bronze Age to modern sheep, with no obvious interruptions, introgressions or imports from outside regions and in spite of the introduction of new breeds from the 18^th^ century onwards. Therefore, it seems that sheep husbandry in the Estonian area has been persistent and relatively autochthonous. Even if human-mediated animal movements did occur, these did not affect the majority of the sheep population, as demonstrated by a lack of significant genetic differentiation between the ancient populations. Nevertheless, some changes in maternal genetic diversity were observed in the first half of the Middle Ages when sheep populations seem to have expanded due to the growing human populations and their need for subsistence. Further investigation is needed to determine the extent to which changes in the animals’ physical size is related to this population expansion or to other environmental and climatic changes. We also explored three micro-regions to examine sheep husbandry in detail, supporting the changes seen in the 13^th^ century, as predominantly new haplotypes were observed in medieval Viljandi-Karksi, Tartu-Lohkva and Saaremaa Island compared to the Late Iron Age. Due to relatively small sample sizes, questions of continuity at a micro-scale remain open.

A significant change was recorded in the modern Kihnu native sheep, which had gone through a population decrease during the last centuries, resulting in lower haplotype diversity and high rate of novel haplotypes compared to the ancient populations. Nevertheless, our results suggest a clear connection between the earliest studied sheep and Kihnu native breed, and induce further research on their history and position among the northern European native sheep breeds.

As studies of short mitochondrial DNA fragments have limitations, phenotypic and Y-chromosome DNA needs to be studied, especially by improved aDNA extraction and high-throughput sequencing. Further genetic, archaeological, and historical studies of modern and ancient sheep in Estonia and neighbouring regions would expand the understanding of both the early stages of the first domesticates in the region as well as the subsequent development of the populations.

## Supporting Information

S1 Fig Median-joining network of ancient and modern samples from this study and Finland. (PDF)

S1 Table Sample data. (PDF)

S2 Table List of primers. (PDF)

S3 Table Success rate of ancient samples. (PDF)

S4 Table Population pairwise *F*_*ST*_ of four temporal cohorts of Estonian ancient and modern Kihnu native sheep. (PDF)

S5 Table Haplotype data for ancient and modern samples of this study. (PDF)

S6 Table Haplotype data for ancient and modern samples of this study, and for comparative samples from Finland. (PDF)

S1 Text Sampling and DNA extraction protocol. (PDF)

S2 Text Radiocarbon dating. (PDF)

## References

[1] Zeder MA. Domestication and early agriculture in the Mediterranean Basin: origins, diffusion, and impact. Proc Natl Acad Sci U S A. 2008;105(33):11597–11604. 10.1073/pnas.0801317105 18697943 PMC2575338

[2] Ryder ML. Sheep and Man London: Duckworth; 1983.

[3] Chessa B, Arnaud F, Kao RR, Stear MJ, Palmarini M, Alberti A, et al Revealing the history of sheep domestication using retrovirus integrations. Science. 2009;324(5926):532–536. 10.1126/science.1170587 19390051 PMC3145132

[4] Lõugas L, Kriiska A, Maldre L. New dates for the Late Neolithic Corded Ware Culture burials and early husbandry in the East Baltic region. Archaeofauna. 2007;16:21–31.

[5] Lõugas L. Subfossil vertebrate fauna of Asva site, Saaremaa: Mammals. Stilus: Eesti Arheoloogiaseltsi teated. 1994;5:71–93.

[6] Heinrici Chronicon Livoniae / Henriku Liivimaa kroonika. Kleis R, translator. Tarvel E, editor. Tallinn: Tänapäev; 2005.

[7] Rannamäe E, Lõugas L. Animal exploitation in Karksi and Viljandi during the Late Prehistoric Times and the Middle Ages. In: Pluskowski AG, editor. The Ecology of Crusading, Colonisation and Religious Conversion in the Medieval Eastern Baltic: Terra Sacra II. Leiden: Brepols; forthcoming.

[8] Kijas JW, Lenstra JA, Hayes B, Boitard S, Porto Neto LR, San Cristobal M, et al Genome-wide analysis of the world's sheep breeds reveals high levels of historic mixture and strong recent selection. PloS Biol. 2012;10(2):e1001258 10.1371/journal.pbio.1001258 22346734 PMC3274507

[9] Lancioni H, Di Lorenzo P, Ceccobelli S, Perego UA, Miglio A, Landi V, et al Phylogenetic Relationships of Three Italian Merino-Derived Sheep Breeds Evaluated through a Complete Mitogenome Analysis. PLoS ONE. 2013;8(9):e73712 10.1371/journal.pone.0073712 24040036 PMC3767607

[10] Niemi M, Bläuer A, Iso-Touru T, Nyström V, Harjula J, Taavitsainen JP, et al Mitochondrial DNA and Y-chromosomal diversity in ancient populations of domestic sheep (*Ovis aries*) in Finland: comparison with contemporary sheep breeds. Genet Sel Evol. 2013;45(2). 10.1186/1297-9686-45-2 PMC3558444 23339395

[11] Soom A. Der Herrenhof in Estland im 17 Jahrhundert. Lund: Eesti Teaduslik Selts Rootsis; 1954.

[12] Jaama K. Eesti tumedapealine lambatõug. Tallinn: Eesti Riiklik Kirjastus; 1959.

[13] Luik H, Piirsalu P, Vahejõe K. Lambakasvatuse valdkonna käsiraamat. Tartu: Eesti Maaülikool, Majandus- ja sotsiaalinstituut; 2011.

[14] Piirsalu P. Lambakasvatus I. Tartu: Tartumaa Põllumeeste Liit; 2012.

[15] Viinalass H, Värv S, Piirsalu P, Saveli O. Teadmata päritoluga lammastest. Tõuloomakasvatus. 2006;4:20–23.

[16] Rannamäe E, Lõugas L, Niemi M, Kantanen J, Maldre L, Kadõrova N, et al Maternal and paternal genetic diversity of ancient sheep in Estonia from the Bronze Age to the Post-Medieval Period, and comparison with other regions in Eurasia. Anim Genet. 2016;47(2):208–218. 10.1111/age.12407 26805771

[17] Ćinkulov M, Popovski Z, Porcu K, Tanaskovska B, Hodžić A, Bytyqi H, et al Genetic diversity and structure of the West Balkan Pramenka sheep types as revealed by microsatellite and mitochondrial DNA analysis. J Anim Breed Genet. 2008;125(6):417–426. 10.1111/j.1439-0388.2008.00742.x 19134078

[18] Haak A, Rannamäe E, Luik H, Maldre L. Worked and unworked bone from the Viljandi castle of the Livonian Order (13th–16th centuries). In: Kurila L, editor. Lietuvos Archeologija 38. Vilnius: Lietuvos istorijos institutes; 2012. pp. 295–338.

[19] Haak A, Rannamäe E. Tracing the castle crew. (Zoo)archaeological search for the inhabitants of Viljandi castle (South Estonia) in the late 13th century. In: Predovnik K, editor. Castrum Bene 12. Ljubljana: University of Ljubljana, Faculty of Arts; 2014. pp. 139–152.

[20] Maldre L. Karjakasvatusest Ridala pronksiaja asulas. In: Lang V, editor. Loodus, inimene ja tehnoloogia 2. Muinasaja teadus 17. Tallinn, Tartu: Tallinna Raamatutrükikoda; 2008. pp. 263–276.

[21] Maldre L. Koduloomaluud keskaegsest Tallinnast. In: Lang V, editor. Loodus, inimene ja tehnoloogia 2. Muinasaja teadus 17. Tallinn, Tartu: Tallinna Raamatutrükikoda; 2008. pp. 277–311.

[22] Peets J, Maldre L. Eesti kohaliku lambatõu kujunemisest arheoloogilise ja osteoloogilise materjali põhjal ehk neljasarvelised lambad ning Jakobsoni must kuub. Kleio–Ajaloo Ajakiri. 1995;1(11):3–4.

[23] Rannamäe E. A Zooarchaeological Study of Animal Consumption in Medieval Viljandi. MA Thesis, University of Tartu. 2010. Available: http://www.arheo.ut.ee/theses/Eve_Rannam%E4eMA2010.pdf

[24] Rannamäe E, Valk H. Some spatial and temporal aspects of animal utilisation in Viljandi, Medieval Livonia In: Pluskowski A, Brown A, Stančikaitė M, Daugnora L, editors. Archaeologia Baltica 20. Klaipėda: Klaipėda University; 2013 pp. 47–58.

[25] Rammo R. Tekstiilileiud Tartu keskaegsetest jäätmekastidest: tehnoloogia, kaubandus ja tarbimine / Textile finds from medieval cesspits in Tartu: technology, trade and consumption. PhD Thesis, University of Tartu. 2015. Available: https://dspace.ut.ee/handle/10062/49523?locale-attribute=en

[26] Zeder MA. Reconciling Rates of Long Bone Fusion and Tooth Eruption and Wear in Sheep (*Ovis*) and Goat (*Capra*) In: Ruscillo D, editor. Recent Advances in Ageing and Sexing Animal Bones. Oxford: Oxbow Books; 2006 pp. 87–117.

[27] Yang DY, Eng B, Waye JS, Dudar JC, Saunders SR. Technical note: improved DNA extraction from ancient bones using silica-based spin columns. Am J Phys Anthropol. 1998;105(4):539–543. 10.1002/(SICI)1096-8644(199804)105:4<539::AID-AJPA10>3.0.CO;2-1 9584894

[28] Hiendleder S, Lewalski H, Wassmuth R, Janke A. The complete mitochondrial DNA sequence of the domestic sheep (*Ovis aries*) and comparison with the other major ovine haplotype. J Mol Evol. 1998;47(4):441–448. 9767689 10.1007/pl00006401

[29] Keis M, Remm J, Ho SYW, Davison J, Tammeleht E, Tumanov IL, et al Complete mitochondrial genomes and a novel spatial genetic method reveal cryptic phylogeographic structure and migration patterns among brown bears in north-western Eurasia. J Biogeogr. 2013;40(5):915–927. 10.1111/jbi.12043

[30] Cooper A, Poinar HN. Ancient DNA: Do it right or not at all. Science. 2000;289(5482):1139 10.1126/science.289.5482.1139b 10970224

[31] Poinar HN. The top 10 list: criteria of authenticity for DNA from ancient and forensic samples. Int Congr Ser. 2003;1239:575–579. 10.1016/S0531-5131(02)00624-6

[32] Yang DY, Watt K. Contamination controls when preparing archaeological remains for ancient DNA analysis. J Archaeol Sci. 2005;32(3):331–336. 10.1016/j.jas.2004.09.008

[33] Briggs AW, Stenzel U, Johnson PLF, Green RE, Kelso J, Prüfer K, et al Patterns of damage in genomic DNA sequences from a Neandertal. Proc Natl Acad Sci U S A. 2007;104(37):14616–14621. 10.1073/pnas.0704665104 17715061 PMC1976210

[34] Hofreiter M, Jaenicke V, Serre D, von Haeseler A, Pääbo S. DNA sequences from multiple amplifications reveal artifacts induced by cytosine deamination in ancient DNA. Nucleic Acids Res. 2001;29(23):4793–4799. 10.1093/nar/29.23.4793 11726688 PMC96698

[35] Saarma U, Jõgisalu I, Moks E, Varcasia A, Lavikainen A, Oksanen A, et al A novel phylogeny for the genus *Echinococcus*, based on nuclear data, challenges relationships based on mitochondrial evidence. Parasitology. 2009;136(3):317–328. 10.1017/S0031182008005453 19154654

[36] Kearse M, Moir R, Wilson A, Stones-Havas S, Cheung M, Sturrock S, et al Geneious Basic: an integrated and extendable desktop software platform for the organization and analysis of sequence data. Bioinformatics. 2012;28(12):1647–1649. 10.1093/bioinformatics/bts199 22543367 PMC3371832

[37] Librado P, Rozas J. DnaSP v5: A software for comprehensive analysis of DNA polymorphism data. Bioinformatics. 2009;25(11):1451–1452. 10.1093/bioinformatics/btp187 19346325

[38] Bandelt HJ, Forster P, Röhl A. Median-joining networks for inferring intraspecific phylogenies. Mol Biol Evol. 1999;16(1):37–48. 10.1093/oxfordjournals.molbev.a026036 10331250

[39] Prost S, Anderson CNK. TempNet: a method to display statistical parsimony networks for heterochronous DNA sequence data. Methods Ecol Evol. 2011;2(6):663–667. 10.1111/j.2041-210X.2011.00129.x

[40] R Core Team. R: A language and environment for statistical computing. Vienna: R Foundation for Statistical Computing; 2014.

[41] Reynolds J, Weir BS, Cockerham CC. Estimation of the Co-Ancestry Coefficient–Basis for a Short-Term Genetic-Distance. Genetics. 1983;105(3):767–779. 17246175 10.1093/genetics/105.3.767 PMC1202185

[42] Excoffier L, Lischer HEL. Arlequin suite ver 3.5: A new series of programs to perform population genetics analyses under Linux and Windows. Mol Ecol Resour. 2010;10(3):564–567. 10.1111/j.1755-0998.2010.02847.x 21565059

[43] Bollvåg AØ. Mitochondrial Ewe–application of ancient DNA typing to the study of domestic sheep (Ovis aries) in mediaeval Norway. M.Sc. Thesis, University of Oslo. 2010. Available: https://www.duo.uio.no/handle/10852/11765

[44] Brandt LØ, Tranekjer LD, Mannering U, Ringgaard M, Frei KM, Willerslev E, et al Characterising the potential of sheep wool for ancient DNA analyses. Archaeol Anthropol Sci. 2011;3(2):209–221. 10.1007/s12520-011-0055-2

[45] Olivieri C, Ermini L, Rizzi E, Corti G, Luciani S, Marota I, et al Phylogenetic position of a copper age sheep (*Ovis aries*) mitochondrial DNA. PLoS ONE. 2012;7(3); e33792 10.1371/journal.pone.0033792 22457789 PMC3311544

[46] Rast-Eicher A, Bender Jørgensen L. Sheep wool in Bronze Age and Iron Age Europe. J Archaeol Sci. 2013;40(2):1224–1241. 10.1016/j.jas.2012.09.030

[47] Wood NJ, Phua SH. Variation in the control region sequence of the sheep mitochondrial genome. Anim Genet. 1996;27(1):25–33. 10.1111/j.1365-2052.1996.tb01173.x 8624033

[48] Guo J, Du LX, Ma YH, Guan WJ, Li HB, Zhao QJ, et al A novel maternal lineage revealed in sheep (*Ovis aries*). Anim Genet. 2005;36(4):331–336. 10.1111/j.1365-2052.2005.01310.x 16026344

[49] Lv FH, Peng WF, Yang J, Zhao YX, Li WR, Liu MJ, et al Mitogenomic Meta-Analysis Identifies Two Phases of Migration in the History of Eastern Eurasian Sheep. Mol Biol Evol. 2015;32(10):2515–2533. 10.1093/molbev/msv139 26085518 PMC4576706

[50] Pedrosa S, Uzun M, Arranz JJ, Gutiérrez-Gil B, San Primitivo F, Bayón Y. Evidence of three maternal lineages in Near Eastern sheep supporting multiple domestication events. Proc Biol Sci. 2005;272(1577):2211–2217. 10.1098/rspb.2005.3204 16191632 PMC1559946

[51] Meadows JRS, Cemal I, Karaca O, Gootwine E, Kijas JW. Five ovine mitochondrial lineages identified from sheep breeds of the near East. Genetics. 2007;175(3):1371–1379. 10.1534/genetics.106.068353 17194773 PMC1840082

[52] Tapio M, Marzanov N, Ozerov M, Ćinkulov M, Gonzarenko G, Kiselyova T, et al Sheep Mitochondrial DNA Variation in European, Caucasian, and Central Asian Areas. Mol Biol Evol. 2006;23(9):1776–1783. 10.1093/molbev/msl043 16782761

[53] Cai DW, Han L, Zhang XL, Zhou H, Zhu H. DNA analysis of archaeological sheep remains from China. J Archaeol Sci. 2007;34(9):1347–1355. 10.1016/j.jas.2006.10.020

[54] Cai DW, Tang Z, Zhu H, Zhou H, Yu H, Han L, et al Early history of Chinese domestic sheep indicated by ancient DNA analysis of Bronze Age individuals. J Archaeol Sci. 2011;38(4):896–902. 10.1016/j.jas.2010.11.019

[55] Demirci S, Koban Baştanlar E, Dağtaş ND, Pişkin E, Engin A, Ozer F, et al Mitochondrial DNA Diversity of Modern, Ancient and Wild Sheep (*Ovis gmelinii anatolica*) from Turkey: New Insights on the Evolutionary History of Sheep. PLoS ONE. 2013;8(12):e81952 10.1371/journal.pone.0081952 24349158 PMC3859546

[56] Gabbianelli F, Gargani M, Pariset L, Mariotti M, Alhaique F, De Minicis E, et al Mitochondrial DNA analysis of medieval sheep (*Ovis aries*) in central Italy reveals the predominance of haplogroup B already in the Middle Ages. Anim Genet. 2015;46(3):329–332. 10.1111/age.12289 25917303

[57] Horsburgh KA, Rhines A. Genetic characterization of an archaeological sheep assemblage from South Africa’s Western Cape. J Archaeol Sci. 2010;37(11):2906–2910. 10.1016/j.jas.2010.06.035

[58] Kahila Bar-Gal G, Ducos P, Horwitz LK. The application of ancient DNA analysis to identify Neolithic caprinae: A case study from the site of Hatoula, Israel. Int J Osteoarchaeol. 2003;13(3):120–131. 10.1002/oa.666

[59] Lawson Handley LJ, Byrne K, Santucci F, Townsend S, Taylor M, Bruford MW, et al Genetic structure of European sheep breeds. Heredity. 2007;99(6):620–631. 10.1038/sj.hdy.6801039 17700634

[60] Peter C, Bruford M, Perez T, Dalamitra S, Hewitt G, Erhardt G, et al Genetic diversity and subdivision of 57 European and Middle-Eastern sheep breeds. Anim Genet. 2007;38(1):37–44. 10.1111/j.1365-2052.2007.01561.x 17257186

[61] Tapio M, Ozerov M, Tapio I, Toro MA, Marzanov N, Ćinkulov M, et al Microsatellite based genetic diversity and population structure of domestic sheep in northern Eurasia. BMC Genet. 2010;11(76). 10.1186/1471-2156-11-76 PMC2931448 20698974

[62] Niemi M, Bläuer A, Iso-Touru T, Harjula J, Nyström-Edmark V, Rannamäe E, et al Temporal Fluctuation in North East Baltic Sea Region Cattle Population Revealed by Mitochondrial and Y-Chromosomal DNA Analyses. PloS ONE. 2015;10(5):e0123821 10.1371/journal.pone.0123821 25992976 PMC4439080

[63] Goslar T, Kalicki T, Kaczanowska M, Kozłowski JK. Stratigraphic sequence in trench A: complex II, layers 2–12 –from the Early Neolithic to the Palaeolithic. In: Kaczanowska M, Kozłowski JK, Sampson A, editors. The Sarakenos Cave at Akraephnion, Boeotia, Greece. Vol. II. The Early Neolithic, the Mesolithic and the Final Palaeolithic (Excavations in Trench A). Kraków: The Polish Academy of Arts and Sciences; 2016. pp. 18–33.

[64] Hadjigeorgiou I. Past, present and future of pastoralism in Greece. Pastoralism. 2011;1(24). 10.1186/2041-7136-1-24

[65] Wilczyński J, Tomek T, Pryor A. Archaeozoological record In: Kaczanowska M, Kozłowski JK, Sampson A, editors. The Sarakenos Cave at Akraephnion, Boeotia, Greece. Vol. II. The Early Neolithic, the Mesolithic and the Final Palaeolithic (Excavations in Trench A). Kraków: The Polish Academy of Arts and Sciences; 2016 pp. 81–90.

[66] Valk H, Rannamäe E, Brown AD, Pluskowski A, Badura M, Lõugas L. Thirteenth century cultural deposits at the castle of the Teutonic Order in Karksi. Archaeological Fieldwork in Estonia / Arheoloogilised välitööd Eestis 2012 2013:73–92.

[67] Palli H. Eesti rahvastiku ajalugu aastani 1712 Academia 6. Tallinn: Teaduste Akadeemia Kirjastus; 1996.

[68] Palli H. Eesti rahvastiku ajalugu 1712–1799 Academia 7. Tallinn: Teaduste Akadeemia Kirjastus; 1997.

[69] Karelson M. Lehekülgi Eesti põllumajanduse ja talurahva minevikust Tallinn: Valgus; 1981.

[70] Michelson A. Kiltsi niidul eesti maalammaste vabapidamisel saadud kogemused In: Kastanje V, editor. Traditsiooniline lambakasvatus Eesti ja Soome rannikualadel ning saartel. Tallinn: Eesti Taimekasvatuse Instituut; 2013 pp. 60–94.

[71] Tajima F. Statistical Method for Testing the Neutral Mutation Hypothesis by DNA Polymorphism. Genetics. 1989;123(3):585–595. 2513255 10.1093/genetics/123.3.585 PMC1203831

[72] Pluskowski A, Valk H. Conquest and Europeanisation: the archaeology of the crusades in Livonia, Prussia and Lithuania In: Boas AJ, editor. The Crusader World. London, New York: Routledge; 2016 pp. 568–592.

[73] Pluskowski A, Brown A, Banerjea R, Makowiecki D, Seetah K, Rannamäe E, et al From the convent to the commandery: The pivotal role of the environment in defining the medieval Baltic *Ordensland* In: Das Leben im Ordenshaus. Quellen und Studien zur geschichte des Deutschen Ordens. Marburg: Elwert Verlag; forthcoming.

[74] Bruns F, Weczerka H. Hansische Handelsstrassen Teil 2: Textband. Weimar: Böhlaus Nachfolger; 1967.

