# Supplementary material for: Three Thousand Years of Continuity in the Maternal Lineages of Ancient Sheep (Ovis aries) in Estonia
Source: PLoS One. 2016 Oct 12;11(10):e0163676. doi: 10.1371/journal.pone.0163676 (PMC5061334; doi:10.1371/journal.pone.0163676)
Supplement: S1 Fig — (PDF) [file pone.0163676.s001.pdf]

S1 Fig. Median-joining network of ancient and modern samples from this study and Finland. The elaborated time period specific median-joining network of 523 bp mtDNA D-loop haplotypes depicting the relationships between Estonian, Latvian, Russian, Polish, Greek, and Finnish ancient and modern sheep. The samples in the network are: Estonian (*n* = 88), Latvian (*n* = 5), Russian (*n* = 6), Polish (*n* = 2), and Greek (*n* = 1) ancient, and Estonian modern Kihnu native sheep (*n* = 44) of our study; and Finnish ancient (*n* = 26) and modern (*n* = 32) samples from [1]. The numbers of the haplotypes are according to S6 Table. The size of the given node is proportional to the number of samples represented in a haplotype, with the smallest node representing a single individual. Branch length is proportional to the mutational distance; only mutational distances greater than 1 are indicated.

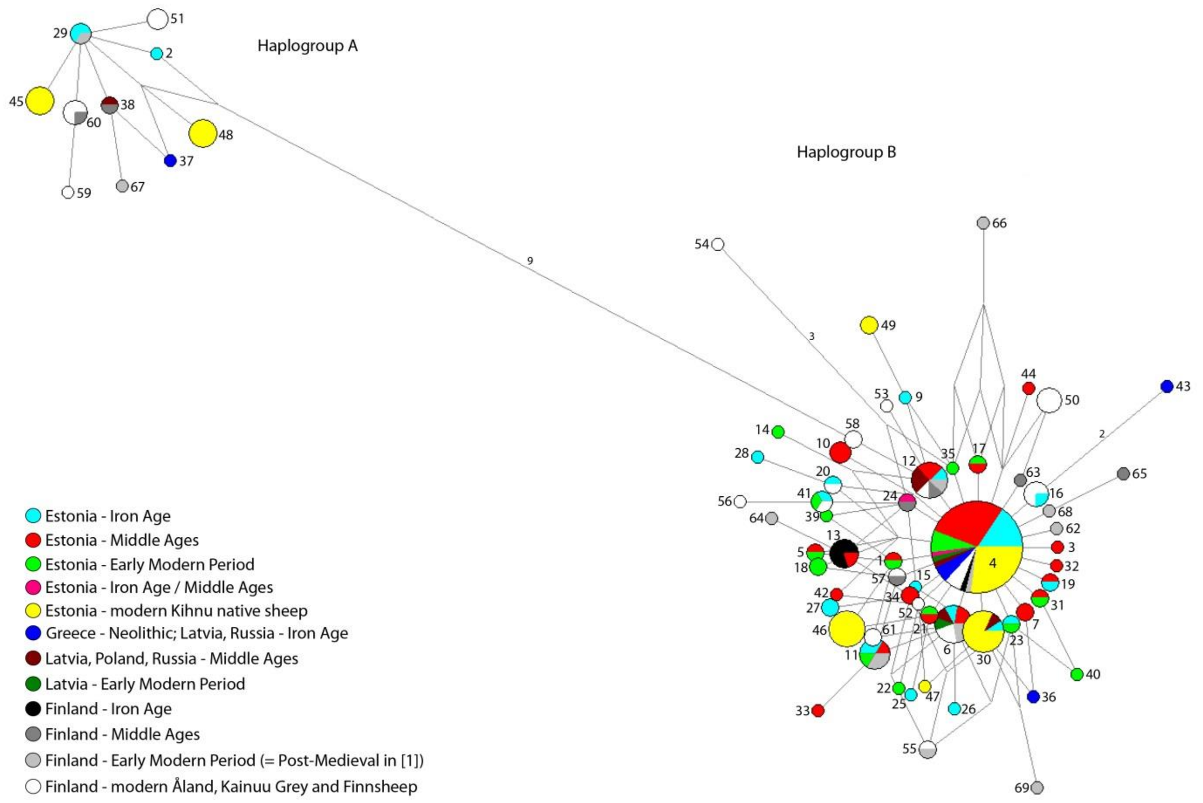

References

1. Niemi M, Bläuer A, Iso-Touru T, Nyström V, Harjula J, Taavitsainen JP, et al. Mitochondrial DNA and Y-chromosomal diversity in ancient populations of domestic sheep (*Ovis aries*) in Finland: comparison with contemporary sheep breeds. Genet Sel Evol. 2013;45(2). doi:10.1186/1297-9686-45-2
