# Supplementary material for: Three Thousand Years of Continuity in the Maternal Lineages of Ancient Sheep (Ovis aries) in Estonia
Source: PLoS One. 2016 Oct 12;11(10):e0163676. doi: 10.1371/journal.pone.0163676 (PMC5061334; doi:10.1371/journal.pone.0163676)
Supplement: S1 Table — (PDF) [file pone.0163676.s002.pdf]

S1 Table. Sample data. Ancient sheep samples used in this study, with relevant archaeological and genetic data. Samples are ordered by time period.

SAMPLE INFORMATION				DATING			SEQUENCING				
**Sample ID**	**Archaeological site, year of excavation, collection code[1]**	**Region (micro-region[2])**	**Skeletal element[3]**	**Dating by archaeological context**	**Dating by AMS ^{14}C**	**Time period[4]**	**No of extract-ions**	**Amplifi-cation success**	**Sequence length (bp)**	**Haplo-group**	**GenBank accession number[5]**
88OaSara1	Sarakenos cave, Greece (no code given)	Greece	atlas	*c.* 6800 BC [1]	-	Initial Neolithic	1	-	-	-	-
123OaSara2	Sarakenos cave, Greece (no code given)	Greece	atlas	*c.* 6800 BC [1]	-	Initial Neolithic	1	+	558	B	KU670303
33OaJoe1	Jõelähtme stone-cist grave 1983 (AI 5306)	Estonia	humerus	1200–800 BC [2]	-	Middle Bronze Age	2**	-	-	-	-
1OaRid1	Ridala settlement site 1961 (AI 4261)	Estonia	humerus	800–600 BC [3]	-	Late Bronze Age	2*	-	213 [4]	B [4]	KP052792 [4]
13OaRid2	Ridala settlement site 1961 (AI 4261)	Estonia (c)	metatarsus	800–600 BC [3]	-	Late Bronze Age	2(*)	+	558	B	KP052801
18OaAsva1	Asva settlement site 1965 (AI 4366)	Estonia (c)	metacarpus	800–600 BC [3]	786–522 BC (95.4%) (2505 ± 30 BP) Lab no: Poz-58805 [4]	Late Bronze Age	2(*)	+	558	B	KP052805
104OaAsva2	Asva settlement site 2013 (AI 7065)	Estonia	mandible	800–600 BC [3]	-	Late Bronze Age	1	-	-	-	-
105OaAsva3	Asva settlement site 2013 (AI 7065)	Estonia (c)	mandible	800–600 BC [3]	-	Late Bronze Age	1	+	558	A	KU670286
35OaKaa1	Kaali settlement site 1977 (AI 5043)	Estonia	proximal phalanx	700–500 BC [3,5–6]	-	Late Bronze Age / Early Iron Age	2**	±	418	B	KU670236
48OaKaa3	Kaali settlement site 1977 (AI 5043)	Estonia	proximal phalanx	700–500 BC [3,5–6]	-	Late Bronze Age / Early Iron Age	1	±	418	B	KU670243
36OaVao1	Väo Jaani stone-cist grave 1982 (AI 5220)	Estonia	metatarsus	500–250 BC [3,7]	-	Early Iron Age	2**	-	-	-	-
55OaVao4	Väo Jaani stone-cist grave 1982 (AI 5220)	Estonia	metatarsus	500–250 BC [3,7]	-	Early Iron Age	1	-	-	-	-
57OaTou1	Tõugu II *tarand* grave 1994 (AI 6003)	Estonia	humerus	250 BC – AD 50 [3]	362–169 BC (95.4%) (2182 ± 31 BP) Lab no: SUERC-55405	Early Iron Age	1	+	558	B	KU670250
61OaAlt1	Alt-Laari settlement site 2008 (TÜ 1695)	Estonia	mandible	AD 50–550 [8]	-	Early Iron Age	1	-		-	-
74OaTou2	Tõugu II *tarand* grave 1995 (AI 6003)	Estonia	radius	500 BC – AD 50 [3]	-	Early Iron Age	1	-		-	-
92OaSpie1	Spietiņi settlement site 1963 (VI 65)	Latvia	humerus	AD 0–300 [9]	-	Early Iron Age	1	±	418	B	KU670276
93OaKivt1	Kivti settlement site 1958 (VI 37)	Latvia	calcaneus	AD 0–600 [10]	AD 125–258 (90.8%) AD 285–287 (0.2%) AD 296–322 (4.3%) (1817 ± 29 BP) Lab no: SUERC-55406	Early Iron Age	1	+	558	A	KU670277
97OaTou3	Tõugu II *tarand* grave 1993 (AI 6003)	Estonia	proximal phalanx	End of Pre-Roman Iron Age [3]	-	Early Iron Age	1	-	-	-	-
107OaMuuk1	Muuksi stone-cist grave 1976 (AI 4980)	Estonia	proximal phalanx	500 BC – AD 550 [3]	AD 180–185 (0.4%) AD 214–386 (95.0%) (1754 ± 31 BP) Lab no: SUERC-55411	Early Iron Age	1	+	558	B	KU670288
23OaRou1	Rõuge settlement site 1959 (AI 4100)	Estonia	metatarsus	AD 500–1000 [11]	AD 666–821 (93.2%) AD 842–860 (2.2%) (1265 ± 30 BP) Lab no: Poz-58804 [4]	Late Iron Age	2(*)	+	558	B	KP052809
25OaSal1	Salme boat-grave 2008 (SM 10601)	Estonia (c)	humerus	AD 700 [12]	-	Late Iron Age	2(*)	+	558	B	KU670233
5OaPada1	Pada settlement site 1981 (AI 5200)	Estonia	metatarsus	AD 700–1100 [13]	-	Late Iron Age	2(*)	+	558	A	KP052794
7OaSoon1	Soontagana hillfort 1968 (PäMu 2/A 2434)	Estonia	metatarsus	AD 900–1200 [14]	-	Late Iron Age	2(*)	+	558	B	KP052796
31OaKivi2	Viljandi settlement site 1999 (VM 10742)	Estonia (a)	radius	AD 1000–1225 [15]	-	Late Iron Age	2(*)	+	558	B	KP052815
30OaKea1	Keava hillfort 2001 (TÜ 1026:419)	Estonia	metatarsus	AD 1000–1300 [16]	-	Late Iron Age	2(*)	+	558	B	KP052814
16OaLoh1	Lõhavere hillfort 1940 (AI 3578:1824)	Estonia	metatarsus	AD 1200–1300 [14]	-	Late Iron Age	2(*)	+	558	B	KP052804
44OaIru1	Iru hillfort 1985 (AI 5302)	Estonia	humerus	Late Iron Age [17]	-	Late Iron Age	1	+	558	B	KU670239
45OaOte2	Otepää settlement site 1989 (AI 5907)	Estonia	humerus	AD 900–1400 [18]	AD 1184–1275 (95.4%) (800 ± 30 BP) Lab no: Poz-61915	Late Iron Age[5]	1	+	558	B	KU670240

49OaIru2	Iru hillfort 1986 (AI 5302)	Estonia	metatarsus	Late Iron Age [17]	-	Late Iron Age	1	+	559	B	KU670244
52OaSuu2	Viljandi settlement site 1999 (VM 10741)	Estonia (a)	tibia	AD 1150–1225 [15]	-	Late Iron Age	1	+	558	B	KU670247
53OaIru3	Iru hillfort 1986 (AI 5302)	Estonia	proximal phalanx	Late Iron Age [17]	-	Late Iron Age	1	+	558	B	KU670248
56OaJoe4	Jõelähtme stone-cist grave 1983 (AI 5306)	Estonia	humerus	1200–800 BC [2]	AD 691–749 (24.7%)<br>AD 762–885 (70.7%)<br>(1224 ± 28 BP)<br>Lab no: SUERC-55404	Late Iron Age	1	+	558	B	KU670249
58OaTor1	Tornimäe settlement site 2004 (AI 6688)	Estonia (c)	metatarsus	AD 800–1050 [19]	-	Late Iron Age	1	+	558	B	KU670251
59OaLin1	Linnaaluste III settlement site 2002 (TÜ 1115:123)	Estonia	metatarsus	AD 700–1100 [20]	-	Late Iron Age	1	+	558	B	KU670252
60OaAak1	Aakre Kivivare hillfort 2011 (TÜ 1928)	Estonia	mandible	AD 550–1050 [21]	-	Late Iron Age	1	-	-	-	-
62OaPada2	Pada I hillfort 1983 (AI 5249)	Estonia	radius	AD 1000–1200 [22]	-	Late Iron Age	1	+	558	B	KU670253
63OaVar2	Varbola Jaanilinn hillfort 1977 (AI 4783)	Estonia	talus	AD 1100–1300 [23]	-	Late Iron Age	1	-	-	-	-
64OaPoi1	Pöide hillfort 1993 (SM 1460)	Estonia (c)	humerus	AD 700–900 / 1100–1300 [24]	-	Late Iron Age	1	+	558	A	KU670254
66OaUug1	Uugla settlement site 2006 (AM A 1026)	Estonia	medial phalanx	AD 1000–1300 [25]	-	Late Iron Age	1	-	-	-	-
71OaKurev1	Kurevere stone grave, year unknown (AI 1394)	Estonia	humerus	Iron Age [3,26]	-	Iron Age	1	-	-	-	-
75OaAak2	Aakre Kivivare hillfort 2011 (TÜ 1928)	Estonia	tooth	AD 800–1050 [21]	-	Late Iron Age	1	±	509	A	KU670260
90OaTer1	Tērvete hillfort 1958 (VI 24e)	Latvia	metacarpus	AD 1000–1200 [27]	-	Late Iron Age	1	+	558	A	KU670274
91OaTart2	Tartu town 2011 (TM A 194)	Estonia (b)	mandible	Iron Age [28]	-	Iron Age	1	+	558	B	KU670275
94OaMez1	Mežmalas hillfort 1963 (VI 80)	Latvia	humerus	AD 600–900 [29]	-	Late Iron Age	1	-	-	-	-
106OaIlm1	Ilmandu III *tarand* grave 1994 (AI 6009)	Estonia	humerus	500 BC – AD 550 [3]	AD 1039–1110 (46.8%)<br>AD 1115–1207 (46.8%)<br>(902 ± 26 BP)<br>Lab no: SUERC-55410	Late Iron Age	1	+	558	B	KU670287
108OaVob1	Võbutõ settlement site 2013 (no code given)	Russia	humerus	AD 1100–1200 [30]	-	Late Iron Age	1	-	-	-	-
109OaMusu1	Viljandi settlement site 2004 (VM 10952)	Estonia (a)	humerus	Viking Age [31]	-	Late Iron Age	1	+	558	B	KU670289
118OaMusu2	Viljandi settlement site 2004 (VM 10952)	Estonia (a)	humerus	Iron Age [31]	AD 1033–1191 (93.7%)<br>AD 1198–1205 (1.7%)<br>(910 ± 31 BP)<br>Lab no: SUERC-55412	Late Iron Age	1	+	558	B	KU670298
119OaRus1	Staraya Ladoga 2010 (no code given)	Russia	metacarpus	AD 870–880 [32]	-	Late Iron Age	1	+	558	B	KU670299
120OaRus2	Staraya Ladoga 2011 (no code given)	Russia	metacarpus	AD 1000–1300 [32]	-	Late Iron Age	1	+	558	B	KU670300
121OaRus3	Staraya Ladoga 2011 (no code given)	Russia	metatarsus	AD 800–1100 [32]	-	Late Iron Age	1	+	558	B	KU670301
133OaKurev2	Kurevere stone grave, year unknown (AI 1394)	Estonia	talus	Iron Age [3,26]	-	Iron Age	2**	-	-	-	-
139OaIlm2	Ilmandu III *tarand* grave 1994 (AI 6009)	Estonia	proximal phalanx	Iron Age [3]	-	Iron Age	1	-	-	-	-
140OaJak3	Tartu town 2011 (TM A 188)	Estonia (b)	metatarsus	AD 1000–1100 [33]	-	Late Iron Age	1	+	558	B	KU670316
65OaPaa1	Paatsa hillfort 1963 (AI 4337)	Estonia (c)	radius	Late Iron Age [34]	-	Late Iron Age	1	+	558	B	KU670255
51OaVar1	Varbola Jaanilinn hillfort 1941 (no code given)	Estonia	humerus	Late Iron Age / Middle Ages [14]	-	Late Iron Age / Middle Ages	1	+	558	B	KU670246
67OaOte3	Otepää hillfort 1961 (AI 4036)	Estonia	metatarsus	AD 1000–1400 [35]	-	Late Iron Age / Middle Ages	1	+	558	B	KU670256
4OaJaan1	Viljandi town 1990 (VM 10258)	Estonia (a)	metatarsus	AD 1250–1300 [36]	-	Middle Ages	2(*)	+	558	B	KP052793
6OaPost1	Viljandi town 2001 (VM 10872)	Estonia (a)	metatarsus	AD 1400–1550 [37]	-	Middle Ages	2(*)	+	558	B	KP052795
9OaSpo1	Viljandi town 1999 (VM 11090)	Estonia (a)	metacarpus	AD 1300–1550 [38]	-	Middle Ages	2(*)	+	558	B	KU670230
10OaHuv1	Viljandi town 1991 (no code given)	Estonia (a)	metacarpus	AD 1225–1350 [39]	-	Middle Ages	2(*)	+	558	B	KP052798
11OaLoss1	Viljandi town 2001 (VM 10848)	Estonia (a)	metatarsus	*c.* AD 1500 [40]	-	Middle Ages	2*	+	558	B	KP052799
12OaOrdu2	Viljandi castle 2003 (VM 10922)	Estonia (a)	calcaneus	AD 1250–1350 [41]	-	Middle Ages	2(*)	+	558	B	KP052800
14OaJaan2	Viljandi town 1990 (VM 10258)	Estonia (a)	metacarpus	AD 1250–1300 [36]	-	Middle Ages	2*	+	558	B	KP052802

15OaSpo2	Viljandi town 1999 (VM 11090)	Estonia (a)	metatarsus	AD 1300–1550 [38]	-	Middle Ages	2(*)	+	558	B	KP052803
17OaOte1	Otepää hillfort 1962 (AI 4036)	Estonia	radius	Middle Ages [42]	-	Middle Ages	2(*)	+	558	B	KU670231
19OaTart1	Tartu town 2011 (TM A 194)	Estonia (b)	humerus	Middle Ages [28]	-	Middle Ages	2(*)	+	558	B	KP052806
20OaTal1	Tallinn town 2008 (AI 6917)	Estonia	metatarsus	AD 1250–1400 [43]	-	Middle Ages	2(*)	+	558	B	KU670232
21OaPar1	Pärnu town 1992 (PäMu 14640/A 2509)	Estonia	cranium	AD 1400–1550 [44]	-	Middle Ages	2(*)	+	558	B	KP052807
22OaPar2	Pärnu town 1992 (PäMu 14640/A 2509)	Estonia	cranium	AD 1400–1550 [44]	-	Middle Ages	2*	-	213 [4]	A [4]	KP052808 [4]
27OaLut1	Tartu town 2009 (TM A 178)	Estonia (b)	metatarsus	AD 1250–1400 [45]	-	Middle Ages	2(*)	+	558	B	KP052811
28OaJak1	Tartu town 2010 (TM A 188)	Estonia (b)	radius	AD 1300–1500 [46]	-	Middle Ages	2(*)	+	558	B	KP052812
29OaJak2	Tartu town 2011 (TM A 188)	Estonia (b)	metacarpus	AD 1250–1500 [33]	-	Middle Ages	2(*)	+	558	B	KP052813
38OaKir1	Kirumpää castle 2005 (TÜ 1433)	Estonia	tibia	AD 1250–1400 [47]	-	Middle Ages	2**	-	-	-	-
43OaLih1	Lihula town 2012 (AM A 1121)	Estonia	radius	AD 1250–1400 [48]	-	Middle Ages	1	+	558	B	KU670238
50OaKir2	Kirumpää settlement site 2005 (TÜ 1434)	Estonia	radius	AD 1250–1700 [47]	AD 1294–1406 (95.4%) (610 ± 35 BP) Lab no: Poz-61910	Middle Ages	1	+	558	B	KU670245
68OaLohk1	Lohkva settlement site 2012 (TÜ 2004)	Estonia	metatarsus	Middle Ages [49]	-	Middle Ages	1	±	418	B	KU670257
69OaHaa1	Haapsalu town 2002 (HM 8914)	Estonia	metacarpus	Middle Ages [50]	-	Middle Ages	1	+	558	B	KU670258
72OaLih2	Lihula town 2012 (AM A 1121)	Estonia	mandible	Middle Ages [48]	-	Middle Ages	1	-	-	-	-
73OaKir4	Kirumpää settlement site 2005 (TÜ 1434)	Estonia	mandible	AD 1250–1700 [47]	-	Middle Ages	1	-	-	-	-
76OaKar1	Karksi castle 2012 (TÜ 1929)	Estonia (a)	humerus	AD 1250–1300 [51]	-	Middle Ages	1	+	558	B	KU670261
77OaVilMu1	Viljandi town 1995 (VM 10942)	Estonia (a)	metatarsus	AD 1250 – beginning of 1300s [52]	-	Middle Ages	1	+	558	B	KU670262
78OaKures1	Kuressaare castle 2012 (SM 10663)	Estonia (c)	humerus	Middle of AD 1300s [53]	-	Middle Ages	1	+	558	B	KU670263
79OaEka1	Tallinn town 2012 (AI 7032:B55)	Estonia	metatarsus	AD 1300–1500 [54]	-	Middle Ages	1	+	558	B	KU670264
80OaKak1	Käku smithy site 2012 (AI 6845)	Estonia (c)	metacarpus	Middle of AD 1400s [55]	-	Middle Ages	1	+	558	B	KU670265
82OaSarg1	Sargvere settlement site 2007 (TÜ 1574)	Estonia	radius	AD 1530–1550 [56]	-	Middle Ages	1	+	558	B	KU670267
83OaPadi1	Padise monastery 2010–2011 (no code given)	Estonia	radius	AD 1300–1500 [57]	-	Middle Ages	1	+	558	B	KU670268
86OaSau1	Tallinn town 1998 (AI 6332)	Estonia	metatarsus	Middle Ages [58]	-	Middle Ages	1	+	558	B	KU670271
87OaNar1	Narva town 2005 (NLM 2420)	Estonia	radius	End of AD 1200s – beginning of 1600s [59]	-	Middle Ages	1	+	558	B	KU670272
95OaVec1	Vecdole castle 1967 (VI 123)	Latvia	metatarsus	AD 1250–1350 [27]	-	Middle Ages	1	+	558	B	KU670278
96OaPih1	Pskov town 2013 (no code given)	Russia	tibia	AD 1300–1500 [60]	-	Middle Ages	1	+	558	A	KU670279
98OaEka2	Tallinn town 2012 (AI 7032:B-63)	Estonia	humerus	AD 1400–1450 [54]	-	Middle Ages	1	±	469	A	KU670280
102OaHar1	Tallinn town 1989 (AI 6176)	Estonia	cranium	Middle Ages [61]	-	Middle Ages	1	+	558	B	KU670284
103OaRoos1	Tallinn town 1996 (AI 6109)	Estonia	cranium	AD 1300–1500 [62]	-	Middle Ages	1	+	558	B	KU670285
110OaBot1	Tartu town 1989 (TM 2032)	Estonia (b)	metatarsus	End of AD 1200s – beginning of 1300s [63]	-	Middle Ages	1	+	558	B	KU670290
111OaLatte1	Tartu town 1987 (TM A 108)	Estonia (b)	humerus	AD 1300–1400 [64]	-	Middle Ages	1	+	558	B	KU670291
112OaLatte2	Tartu town 1987 (TM A 108)	Estonia (b)	metatarsus	AD 1400–1500 [64]	-	Middle Ages	1	+	558	B	KU670292
113OaJaan3	Viljandi town 1991 (VM 10324)	Estonia (a)	metacarpus	AD 1500–1550 [36]	-	Middle Ages	1	+	558	B	KU670293
116OaKrak1	Kraków town 2006 (1822/06)	Poland	radius	AD 1250–1350 [65]	-	Middle Ages	1	+	558	B	KU670296
117OaKrak2	Kraków town 2005 (138/05)	Poland	mandible	AD 1000–1200 [65]	-	Middle Ages	1	±	418	B	KU670297
122OaPih2	Pskov town 2013 (no code given)	Russia	humerus	AD 1400–1500 [60]	-	Middle Ages	1	+	558	B	KU670302

125OaPar3	Pärnu town 1992 (PäMu 14640/A 2509)	Estonia	cranium	Middle Ages [44]	-	Middle Ages	1	±	509	B	KU670305
126OaPar4	Pärnu town 2002 (PäMu A 2570)	Estonia	humerus	AD 1300–1600 [66]	-	Middle Ages	1	±	509	A	KU670306
130OaEka3	Tallinn town 2012 (AI 7032:B-60)	Estonia	metacarpus	AD 1500–1550 [54]	-	Middle Ages	1	+	558	B	KU670310
131OaKura1	Tartu town 1999 (TM A 191)	Estonia (b)	metatarsus	Middle Ages [67]	-	Middle Ages	1	+	558	B	KU670311
132OaSau2	Tallinn town 1998 (AI 6332)	Estonia	metatarsus	Middle Ages [58]	-	Middle Ages	1	+	558	B	KU670312
128OaPih3	Pskov town 2013 (no code given)	Russia	metatarsus	End of AD 1300s – beginning of 1400s [60]	-	Middle Ages	1	+	558	B	KU670308
136OaKar4	Karksi castle 2011 (TÜ 1929)	Estonia (a)	metatarsus	AD 1300–1400 [68]	-	Middle Ages	1	+	558	B	KU670313
137OaKar5	Karksi castle 2012 (TÜ 1929)	Estonia (a)	radius	AD 1200–1300 [51]	-	Middle Ages	1	+	558	B	KU670314
138OaKrak3	Kraków town 2005 (105/05)	Poland	metacarpus	AD 1250–1300 [65]	-	Middle Ages	1	+	558	B	KU670315
143OaKar7	Karksi castle 2011 (TÜ 1929)	Estonia (a)	metatarsus	AD 1400–1500 [68]	-	Middle Ages	1	+	558	B	KU670319
8OaVas1	Vastseliina castle 2005 (TÜ 1435)	Estonia	metatarsus	AD 1500–1700 [69]	-	Early Modern Period	2(*)	+	558	B	KP052797
81OaPol1	Põltsamaa castle 1998 (TÜ 714)	Estonia	humerus	End of AD 1500s [70]	-	Early Modern Period	1	+	558	B	KU670266
84OaKil1	Kiltsi manor 2008 (RM 6782/A 160)	Estonia	radius	End of AD 1500s – beginning of 1600s [71]	-	Early Modern Period	1	+	558	B	KU670269
85OaPai1	Paide castle 2011 (TÜ 1924)	Estonia	humerus	AD 1550 – beginning of 1600s [72]	-	Early Modern Period	1	+	558	B	KU670270
89OaAlu1	Alūksne castle 1982 (VI 231)	Latvia	metatarsus	AD 1500–1700 [27]	-	Early Modern Period	1	+	558	B	KU670273
99OaLohk2	Lohkva settlement site 2012 (TÜ 2004)	Estonia (b)	metacarpus	Beginning of Early Modern Period [49]	-	Early Modern Period	1	+	558	B	KU670281
100OaPadi2	Padise monastery 2010–2011 (no code given)	Estonia	humerus	Time of the Livonian War (1558–1583) [57]	-	Early Modern Period	1	+	558	B	KU670282
101OaPadi3	Padise monastery 2010–2011 (no code given)	Estonia	cranium	AD 1600s [57]	-	Early Modern Period	1	+	559	B	KU670283
114OaJaan4	Viljandi town 1991 (VM 10324)	Estonia (a)	metatarsus	AD 1550 – beginning of 1600s [36]	-	Early Modern Period	1	+	558	B	KU670294
115OaVilKv1	Viljandi town 1997 (VM 10589)	Estonia (a)	humerus	AD 1500–1900 [73]	-	Early Modern Period	1	+	558	B	KU670295
124OaOlu2	Olustvere settlement site 1982 (AI 4998)	Estonia	mandible	Early Modern Period [74]	-	Early Modern Period	1	+	558	B	KU670304
127OaPar5	Pärnu town 2002 (PäMu A 2570)	Estonia	metatarsus	AD 1650 – beginning of 1700s [66]	-	Early Modern Period	1	+	559	B	KU670307
129OaAlu2	Alūksne castle 1978 (VI 231)	Latvia	metacarpus	AD 1500–1700 [27]	-	Early Modern Period	1	+	558	B	KU670309
135OaKar3	Karksi castle 2011 (TÜ 1929)	Estonia	humerus	AD 1550 – beginning of 1600s [68]	-	Early Modern Period	1	-	-	-	-
141OaJak4	Tartu town 2011 (TM A 188)	Estonia (b)	humerus	AD 1600–1700 [33]	-	Early Modern Period	1	+	558	B	KU670317
142OaKar6	Karksi castle 2011 (TÜ 1929)	Estonia (a)	metatarsus	AD 1550 – beginning of 1600s [68]	-	Early Modern Period	1	+	558	B	KU670318
24OaOlu1	Olustvere settlement site 1982 (AI 4998)	Estonia	metatarsus	-	AD 1516–1595 (35.0%)<br>AD 1617–1670 (49.9%)<br>AD 1780–1799 (9.2%)<br>AD 1945–1953 (1.3%)<br>(265 ± 30 BP)<br>Lab no: Poz-58806 [4]	Early Modern / Modern Period	2(*)	+	558	B	KP052810
32OaLoo1	Kihelkonna Loona settlement site 1956 (AI 4129)	Estonia (c)	metatarsus	-	AD 1650–1695 (20.2%)<br>AD 1726–1814 (52.5%)<br>AD 1838–1843 (0.5%)<br>AD 1853–1868 (1.5%)<br>AD 1917–… (20.8%)<br>(185 ± 30 BP)<br>Lab no: Poz-61907	Early Modern / Modern Period	2**	+	558	B	KU670234

34OaPro1	Proosa field remains 2009 (TÜ 1770)	Estonia	mandible	1000–500 BC [75]	AD 1644–1700 (21.9%)<br>AD 1720–1819 (47.9%)<br>AD 1832–1880 (6.8%)<br>AD 1915–… (18.9%)<br>(190 ± 40 BP)<br>Lab no: Poz-61908	Early Modern / Modern Period	1	+	558	B	KU670235
37OaSam1	Sammaste stone grave 1989 (VM 10234)	Estonia	humerus	Iron Age [76]	AD 1681–1738 (27.3%)<br>AD 1757–1762 (0.7%)<br>AD 1803–1937 (67.4%)<br>(105 ± 30 BP)<br>Lab no: Poz-61909	Early Modern / Modern Period	1	+	558	B	KU670237
46OaJoe3	Jõelähtme stone-cist grave 1983 (AI 5306)	Estonia	humerus	1200–800 BC [2]	AD 1650–1695 (20.2%)<br>AD 1726–1814 (52.5%)<br>AD 1838–1843 (0.5%)<br>AD 1853–1868 (1.5%)<br>AD 1917–… (20.8%)<br>(185 ± 30 BP)<br>Lab no: Poz-61913	Early Modern / Modern Period	1	+	558	B	KU670241
47OaVao3	Väo Jaani stone-cist grave 1982 (AI 5220)	Estonia	metatarsus	Early Pre-Roman Iron Age [3,7]	AD 1682–1736 (27.1%)<br>AD 1805–1935 (68.3%)<br>(100 ± 30 BP)<br>Lab no: Poz-61914	Early Modern / Modern Period	1	+	558	B	KU670242
70OaToo1	Toodsi Liidva settlement site 2010 (TÜ 1868)	Estonia	metatarsus	-	AD 1957–1963 (21.0%)<br>AD 1974–1985 (74.4%)<br>(130.72 ± 0.55 pMC)<br>Lab no: Poz-61912	Modern Period	2**	±	469	A	KU670259

[1] **Samples were provided by the following institutions:**

Estonia: University of Tartu (TÜ), Tallinn University (AI), Estonian History Museum (AM), Museum of Viljandi (VM), Tartu City Museum (TM), Saaremaa Museum (SM), Pärnu Museum (PäMu), Virumaa Museum (RM), Narva Museum (NLM), and Läänemaa Museum (HM).
All necessary permits were obtained for the described study, which complied with all relevant regulations:
sampling protocols no 4–13, 15–24, 26–50, 52–58 for the samples held in the collections of TÜ (including samples from AI, VM, TM, PäMu and HM);
sampling protocols no 175–232 for samples held in AI (including samples from AM, SM and PäMu).
No permits were required for the samples held in TM, NLM and RM.
Latvia: Latvian National Museum of History (no permits were required for the described study).
Russia: Archaeological Center of Pskov Region, Zoological Institute of Russian Academy of Sciences (no permits were required for the described study).
Poland: Polish Academy of Sciences (no permits were required for the described study).
Greece: Aegean University Rhodes (no permits were required for the described study).

[2] ***Micro-regions yielding sheep remains over a long chronological span were preferentially targeted to identify temporal changes within a site or region, including:***

(a) Viljandi-Karksi – Viljandi town and its surroundings with Late Iron Age, medieval and early modern deposits, to which the nearby Karksi castle was added, with its exceptionally good stratigraphy from the 13th to 17th century;
(b) Tartu-Lohkva – Tartu town with adjacent Lohkva settlement site with samples from the Late Iron Age to the 17th century;
(c) Saaremaa Island as a geographically separated region with samples dating from the Late Bronze Age up to the 19th century.

[3] ***Most samples were bones, while one was of a tooth:***

humeri (n = 31) and metapodii (n = 56) were preferred, but crania (n = 6), mandibles (n = 10), atlases (n = 2), radii (n = 14), tibiae (n = 3), calcanea (n = 2), tali (n = 2), and phalanges (n = 7) were also chosen as these could be distinguished from goats with more confidence.
Taxonomical identification was assigned with the help of the anatomical reference collection of the Department of Archaeology in the University of Tartu, and using the bone atlas by [77].

[4] The Early Modern Period in the current study corresponds to the Post-Medieval period in [4].

[5] **GenBank accession numbers:**

KP052793–KP052807 and KP052809–KP052815 are for the updated 22 sequences reported first in [4]. Two sequences – 1OaRid1 and 22OaPar2 – are reported in [4], but were unsuccessful with rest of the primer pairs used in this study.
KU670230–KU670319 are for the 90 sequences newly reported in this study.

[5] Based on ^{14}C dating the sample 45OaOte2 falls in between the Late Iron Age and Middle Ages, but is analysed in the Bronze/Iron Age cohort.

* Samples extracted twice: at the Natural Resources Institute Finland, Finland and the University of Tartu, Estonia.
(*) Samples extracted twice: at the Natural Resources Institute Finland, Finland and the University of Tartu, Estonia, but amplified with different primer pairs.
** Samples extracted twice at the University of Tartu, Estonia.

References

1. Goslar T, Kalicki T, Kaczanowska M, Kozłowski JK. Stratigraphic sequence in trench A: complex II, layers 2–12 – from the Early Neolithic to the Palaeolithic. In: Kaczanowska M, Kozłowski JK, Sampson A, editors. The Sarakenos Cave at Akraephnion, Boeotia, Greece. Vol. II. The Early Neolithic, the Mesolithic and the Final Palaeolithic (Excavations in Trench A). Kraków: The Polish Academy of Arts and Sciences; 2016. pp. 18–33.
2. Oras E, Lang V, Rannamäe E, Varul L, Konsa M, Limbo-Simovart J, et al. Tracing prehistoric migration: isotope analysis of Bronze and Pre-Roman Iron Age coastal burials in Estonia. Estonian Journal of Archaeology. 2016;20(1). doi:10.3176/arch.2016.1
3. Lang V. The Bronze and Early Iron Ages in Estonia. Estonian Archaeology 3. Tartu: Tartu University Press; 2007.
4. Rannamäe E, Lõugas L, Niemi M, Kantanen J, Maldre L, Kadõrova N, et al. Maternal and paternal genetic diversity of ancient sheep in Estonia from the Bronze Age to the Post-Medieval Period, and comparison with other regions in Eurasia. Anim Genet. 2016;47(2):208–218. doi:10.1111/age.12407
5. Lõugas V. Archaeological research at Kaali meteorite crater. Eesti NSV Teaduste Akadeemia Toimetised. Ühiskonnateadused. 1978;27(1);64–66.
6. Lõugas V. Arcahaeological excavations in the Kaali crater area. Eesti NSV Teaduste Akadeemia Toimetised. Ühiskonnateadused. 1980;29(4);357–360.
7. Laneman M, Lang V, Malve M, Rannamäe E. New data on Jaani stone graves at Väo, northern Estonia. Estonian Journal of Archaeology. 2015:19(2);110–137.
8. Lillak A, Valk H. Rescue Excavations on Alt-Laari Settlement Site, Tartumaa. Archaeological Fieldwork in Estonia / Arheoloogilised välitööd Eestis 2008. 2009:65–71.
9. Atgāzis M. Spietiņu uzkalniņkapi un to vieta Spietiņu un Plāteru senvietu kopā. In: Caune A, Mugurēvičs Ē, Ose I, Vasks A, editors. Arheoloģija un etnogrāfija XXIII. Veltījums seno apģērbu pētniecei arheoloģei Dr. habil. hist. Annai Zariņai 85 gadu jubilejā. Rīga: Latvijas Vēstures Institūta Apgāds; 2006. pp. 16–40.
10. Šnore E. Celtniecības liecības Kivtu apmetnē. In: Arheoloģija un etnogrāfija XII. Apcerējumi par Latvijas teritorijas apmetnēm, celtnēm un tajās atrasto numismātisko materiālu pirmatnējās kopienas un feodālisma periodā. Rīga: Zinātne; 1978.
11. Lillak A, Valk H. Archaeological trial excavations on Rõuge hill fort, South-East Estonia. Archaeological Fieldwork in Estonia / Arheoloogilised välitööd Eestis 2008. 2009:72–81.
12. Peets J, Allmäe R, Maldre L. Archaeological investigations of Pre-Viking Age burial boat in Salme village at Saaremaa. Archeological Fieldwork in Estonia / Arheoloogilised välitööd Eestis 2010. 2011:29–48.
13. Tamla T. = Тамла Т. Селище в Пада. Eesti NSV Teaduste Akadeemia Toimetised. Ühiskonnateadused. 1983;32(4):302–306.
14. Tõnisson E. Eesti muinaslinnad. Muinasaja teadus 20. Tartu-Tallinn: Tartu Ülikooli Kirjastus; 2008.
15. Valk H. Aruanne arheoloogilistest uurimistöödest Viljandis Suusahüppemäe piirkonnas 04.08.–06.09. Excavation report, manuscript in the archaeology archive of the University of Tartu. 1999.
16. Lang V, Tvauri A, Rohtla M. The hill-fort of Keava. Archaeological Fieldwork in Estonia / Arheoloogilised välitööd Eestis 2001. 2002:65–73.
17. Lang V. Aruanne arheoloogilistest kaevamistest Iru linnuse keskvallil 1985. ja 1986.a. Excavation report, manuscript in the archaeology archive of the University of Tartu. 1988.
18. Valk H. Magistraalkraavist väljatõstetud mullavallide sorteerimisest Otepää linnamäe ja kiriku vahel 1989. a. Excavation report, manuscript in the archaeology archive of the University of Tartu. 1989.
19. Mägi M. Viking Age harbour site at Tornimäe, eastern Saaremaa. Archaeological Fieldwork in Estonia / Arheoloogilised välitööd Eestis 2004. 2005:65–75.
20. Konsa M, Kivi K. Village at the foot of the fort: settlement sites I-III at Linnaaluste. Keava – 'The Hand of the Sun'. Estonian Journal of Archaeology Supplementary series. 2012;1:63–91. doi: 10.3176/arch.2012.supv1.04
21. Valk H, Kama P, Olli M, Rannamäe E. Excavations on the hill forts of South-East Estonia: Kõivuküla, Märdi, Truuta and Aakre. Archaeological Fieldwork in Estonia / Arheoloogilised välitööd Eestis 2011. 2012:27–46.
22. Tamla T. = Тамла Т. Первое городище и хронология комплекса археологических памятников в Пада. Eesti NSV Teaduste Akadeemia Toimetised. Ühiskonnateadused. 1984;33(4):360–363.
23. Tõnisson E, Selirand J. = Тыниссон Э., Селиранд Ю. О раскопках городища Варбола. Eesti NSV Teaduste Akadeemia Toimetised. Ühiskonnateadused. 1978;27(4):358–360.
24. Lõugas V, Mägi-Lõugas M. Excavations at Pöide stronghold have been finished. Eesti NSV Teaduste Akadeemia Toimetised. Ühiskonnateadused. 1994;43(4);390–392.
25. Mandel M. Von den Forschungen am Bodendenkmal zu Uugla. Archaeological Fieldwork in Estonia / Arheoloogilised välitööd Eestis 2006. 2007:113–119.
26. Tvauri A. The Migration Period, Pre-Viking Age, and Viking Age in Estonia. Estonian Archaeology 4. Tartu: Tartu University Press; 2012.
27. Caune A, Ose I. Latvijas viduslaiku pilis IV. Latvijas 12. gadsimta beigu – 17. gadsimta vācu piļu leksikons. Rīga: Latvijas vēstures institūta apgāds; 2004.
28. Tvauri A. Aruanne arheoloogilisest järelevalvest Tartus Lossi 36 ja 38 vee- ja kanalisatsioonitrasside ehituse juures 2011. a. Excavation report, manuscript in the archaeology archive of the University of Tartu. 2016.
29. Mugurēvičs Ē. Oliņkalna un Lokstenes pilsnovadi. Rīga: Zinātne; 1977.
30. Harlashov BN. = Харлашов БН. Отчет об археологических раскопках на территории объекта культурного наследия регионального значения "Селище 2-й пол. I тыс.н.э. в Выбутах" Псковского района Псковской области в 2013 г. Псков. Excavation report, manuscript in the research archive of the Institute of Archaeology, Russian Academy of Science. 2014.
31. Rammo R, Veldi M. Archaeological Excavations at Musumägi Hill in Viljandi. Archaeological Fieldwork in Estonia / Arheoloogilised välitööd Eestis 2004. 2005:103–116.
32. Sedov 1985 = Седов ВВ, editor. Средневековая Ладога. Новые археологические открытия и исследования. Ленинград: Издательство "Наука" Ленинградское Отделение; 1985.
33. Malve M, Roog R, Tvauri A. Preliminary results of the rescue excavation in St Mary's churchyard and its surroundings in Tartu 2010–2011. Archaeological Fieldwork in Estonia / Arheoloogilised välitööd Eestis 2011. 2012:137–50.
34. Kustin A. Aruanne Paatsa linnuse kaevamisest 1963. a. Excavation report, manuscript in the archaeology archive of the University of Tartu. 1968.
35. Mäesalu A. Die Burg Otepää als ein Zentrum in Südostestland im 11.–14. Jh. In: Drake K, editor. Castella maris Baltici I. Stockholm: Almqvist & Wiksell International; 1993. pp. 143–148.
36. Valk H. Aruanne arheoloogilistest kaevamistest Viljandi Jaani kiriku kommunikatsioonidetrassil. Excavation report, manuscript in the archaeology archive of the University of Tartu. 1991.
37. Haak A. Aruanne arheoloogilistest päästekaevamistest Viljandis, AS Domotex keskküttetrassi alal Posti tn 16 ja Lossi tn 33 vahelises hoovis 31. oktoobrist 17. novembrini 2001. a. Excavation report, manuscript in the archaeology archive of the University of Tartu. 2002.
38. Tvauri A. Aruanne arheoloogilistest päästekaevamistest Viljandi Spordihoone juurdeehituse alal 1999. aastal. Excavation report, manuscript in the archaeology archive of the University of Tartu. 1999.
39. Valk H. Aruanne arheoloogilistest kaevamistest Viljandi Noorte Huvikeskuse küttetrassil 1991. a. Excavation report, manuscript in the archaeology archive of the University of Tartu. 1993.
40. Haak A, Valk H. Archaeological investigations of medieval and post-medieval Viljandi. Archaeological Fieldwork in Estonia / Arheoloogilised välitööd Eestis 2001. 2002:91–104.
41. Haak A. Archaeological investigations at Viljandi castle of the Teutonic Order and in Medieval Viljandi. Archeological Fieldwork in Estonia / Arheoloogilised välitööd Eestis 2003. 2004:107–122.

42. Jaanits L, Laul S, Lõugas V, Tõnisson E. Eesti esiajalugu. Tallinn: Eesti Raamat; 1982.
43. Kadakas V, Nurk R, Püüa G, Toos G, Lõugas L, Hiie S, et al. Rescue excavations in Tallinn Vabaduse Square and Ingermanland bastion 2008–2009. Archaeological Fieldwork in Estonia / Arheoloogilised välitööd Eestis 2009. 2010:49–72.
44. Lõugas L. Pärnu Malmö tänav 15 arheoloogilised päästekaevamised 1992. Report on the archaeozoological analysis, manuscript in the archive of the Institute of History, Tallinn University. 1992.
45. Kriiska A, Roog R, Alttoa K, Allik A, Läänelaid A, Bernotas R, et al. The medieval and modern period building complex at 2 Lutsu Street in Tartu. Results of the archaeological, architectural historical, dendrochronological and osteoarchaeological research. Ajalooline Ajakiri. 2011;1(135);3–40.
46. Tvauri A. Archaeological investigations at the courtyard of Jakobi Street 2 / Lossi Street 3, Tartu. Archaeological Fieldwork in Estonia / Arheoloogilised välitööd Eestis 2010. 2011:179–186.
47. Valk H. Excavations in the Late Iron Age and medieval Centres of Võrumaa: Tilleoru, Kirumpää and Vastseliina. Archeological Fieldwork in Estonia / Arheoloogilised välitööd Eestis 2005. 2006:127–140.
48. Russow E, Haak A. Archaeological fieldwork in 2012. Archaeological Fieldwork in Estonia / Arheoloogilised välitööd Eestis 2012. 2013:9–28.
49. Roog R, Malve M. Rescue excavations on the settlement site and rural cemetery of Lohkva, Tartumaa. Archaeological Fieldwork in Estonia / Arheoloogilised välitööd Eestis 2012. 2013:241–250.
50. Tvauri A. Aruanne arheoloogilistest kaevamistest Haapsalus Jaani 4 krundil 2002. aastal. Excavation report, manuscript in the archaeology archive of the University of Tartu. 2002.
51. Valk H, Rannamäe E, Brown AD, Pluskowski A, Badura M, Lõugas L. Thirteenth century cultural deposits at the castle of the Teutonic Order in Karksi. Archaeological Fieldwork in Estonia / Arheoloogilised välitööd Eestis 2012. 2013:73–92.
52. Haak A, Rannamäe E. A Household by the Market Place: Archaeological and Zooarchaeological Contributions to the Development of Urban Viljandi, Estonia. In: Viitanen EM, editor. 18th annual meeting, European Association of Archaeologists, 29 August – 1 September 2012 Helsinki, Finland, abstracts. Helsinki: University of Helsinki; 2012. p. 94.
53. Püüa G, Toos G, Alttoa K, Kadakas V. Additions to the early construction history of the Kuressaare bishop's castle. Archeological Fieldwork in Estonia / Arheoloogilised välitööd Eestis 2012. 2013:171–84.
54. Russow E, Lõugas L, Maldre L, Hiie S, Kihno K, Luik H, et al. Medieval and early modern suburban site in Tallinn, Tartu Rd. 1: artefacts and ecofacts. Archaeological Fieldwork in Estonia / Arheoloogilised välitööd Eestis 2012. 2013:149–170.
55. Peets J, Saage R, Maldre L. The medieval and early modern smithy site of Käku. Archaeological Fieldwork in Estonia / Arheoloogilised välitööd Eestis 2012. 2013:93–108.
56. Tvauri A. Archaeologycal investigations at the Iron Age and Medieval settlement site of Sargvere. Archaeological Fieldwork in Estonia / Arheoloogilised välitööd Eestis 2007. 2008:109–114.
57. Lõugas L, Maldre L, Tomek T, Kadakas V. Archaeozoological evidence from the Padise monastery. Archaeological Fieldwork in Estonia / Arheoloogilised välitööd Eestis 2011. 2012:83–92.
58. Tiko A. Keskaegsed majapidamised Tallinnas Sauna tn 8/10 kaevamiste näitel. MA Thesis, Tallinn University. 2014. Available: https://www.academia.edu/7719850/Magistrit%C3%B6%C3%B6_Keskaegsed_majapidamised_Tallinnas_Sauna_tn_8_10_kaevamiste_n%C3%A4itel._
59. Kriiska A, Lõhmus M. Archaeological excavations on Suur street, Narva Town. Archaeological Fieldwork in Estonia / Arheoloogilised välitööd Eestis 2005. 2006:189–206.
60. Ershova E. = Ершова ТЕ. Отчет об археологических раскопках на улице К.Маркса в г.Пскове в 2013 г. Новоторговский -XI раскоп. Excavation report, manuscript in the research archive of the Institute of Archaeology, Russian Academy of Science. 2015.
61. Peets J, Maldre L. Eesti kohaliku lambatõu kujunemisest arheoloogilise ja osteoloogilise materjali põhjal ehk neljasarvelised lambad ning Jakobsoni must kuub. Kleio. Ajaloo ajakiri. 1995;1(11):3–4.
62. Sokolovski V. Aruanne arheoloogilistest uuringutest Tallinnas, Roosikrantsi tn 9 ja 11, I. Uuringute tulemused. Excavation report, manuscript in the archaeology archive of the Tallinn University. 1997.
63. Mäesalu A. Die Haustypen in hansezeitlichen Tartu (Dorpat). In: Lübecker Kolloquium zur Stadtarchäologie im Hanseraum III: der Hausbau / Bereich Archäologie der Hansestadt Lübeck ; herausgegeben von Manfred Gläser. Lübeck: Schmidt-Römhild; 2001. pp. 581–594.
64. Mäesalu A. Sechs Holzkonstruktionen in Tartu (Lossi-Strasse). Eesti Teaduste Akadeemia Toimetised. Ühiskonnateadused. 1990;39(4);446–452.
65. Wojtal P, Makowiecki D, Wertz K, Wilczyński J, Miękina B, Zabilska M. Wstępne wyniki badań zooarcheologicznych szczątków kostnych z wykopalisk prowadzonych na Rynku Krakowskim w latach 2005–2007. In: Firlet E, editor. Krzysztofory. Zeszyty Naukowe Muzeum Historycznego Miasta Krakowa 28, część 2. Kraków: Muzeum Historyczne Miasta Krakowa; 2010. pp. 137–150.
66. Kadakas V, Haak A, Russow E, Saluäär U, Sarv K. Archaeological Investigations in Pärnu. Archaeological Fieldwork in Estonia / Arheoloogilised välitööd Eestis 2002. 2003:179–209.
67. Valk H. Aruanne arheoloogilistest kaevamistest Tartus Kuradisilla juures 1999. a. mais. Excavation report, manuscript in the archaeology archive of the University of Tartu. 1999.
68. Valk H, Pluskowski A, Brown AD, Rannamäe E, Malve M, Varul L. The castle of the Teotonic Order in Karksi: preliminary excavation results. Archaeological Fieldwork in Estonia / Arheoloogilised välitööd Eestis 2011. 2012:47–56.
69. Valk H. Excavations in the ruins of Vastseliina Castle and on the hillforts of Urvaste and Hinniala. Archeological Fieldwork in Estonia / Arheoloogilised Välitööd Eestis 2001. 2007:49–67.
70. Tvauri A. Archaeological excavations in the gate building of Põltsamaa castle. Archaeological Fieldwork in Estonia / Arheoloogilised välitööd Eestis 1998. 1999:127–131.
71. Jonuks T. Kiltsi mõis – keskaegsest kivimajast esindusliku härrastehooneni. SA Virumaa Muuseumid toimetised 2012. 2012:32–47.
72. Tvauri A. Aruanne Paide Vallimäel Kõlakoja rekonstrueerimse ja mänguväljaku rajamise juures 2001. aastal toimunud arheoloogilisest järelvalvest. Excavation report, manuscript in the archaeology archive of the University of Tartu. 2011.
73. Tvauri A. Archaeological investigations in the old part of Viljandi. Archaeological Fieldwork in Estonia / Arheoloogilised välitööd Eestis 1997. 1998:81–86.
74. Lavi A. Aruanne uurimistöödest Olustvere asulakohal 5.09.–15.10.1978. Excavation report, manuscript in the archaeology archive of the Tallinn University. 1978.
75. Kaldre H, Aguraiuja Ü, Livin L. Preliminary investigations of fossil field systems at Loo. Archaeological Fieldwork in Estonia / Arheoloogilised välitööd Eestis 2009. 2010:73–84.
76. Valk H. Aruanne arheoloogilistest kaevamistest Sammaste kivikalmel 26.06.–31.07.1989. a. Excavation report, manuscript in the archaeology archive of the University of Tartu. 1989.
77. Boessneck J, Müller HH, Teichert M. Osteologische Unterscheidungsmerkmale zwischen Schaf (*Ovis aries* LINNÉ) und Ziege (*Capra hircus* LINNÉ). Kühn-Archiv. Arbeiten aus der Landwirtschaftlichen Fakultät der Martin-Luther-Universität Halle-Wittenberg. Band 78, Heft 1/2. Berlin: Akademie-Verlag; 1964.
