# Supplementary material for: Three Thousand Years of Continuity in the Maternal Lineages of Ancient Sheep (Ovis aries) in Estonia
Source: PLoS One. 2016 Oct 12;11(10):e0163676. doi: 10.1371/journal.pone.0163676 (PMC5061334; doi:10.1371/journal.pone.0163676)
Supplement: S2 Table — (PDF) [file pone.0163676.s003.pdf]

S2 Table. List of primers. List of primers used in this study, with fragment length, nucleotide position of initiation of amplification according to GenBank accession NC001941 [1], and success rate of each primer.

		Primer pair	Fragment length	Start position [NC001941]	Success rate	References
Ancient	Fragment 1	For 5’GTTTCACTGAAGCATGTAGGG3’<br>Rev 5’CATGGTGAACAAGCTCGTGA3’	116 bp	15957	88%	[2]
	Fragment 2	For 5’TCAACATGCGTATCCTGTCC3’<br>Rev 5’ATGGCCCTGAAGAAAGAACC3’	164 bp	16027	84%	[2]
	Fragment 3	For 5’CCCATTAACTGTGGGGGTAA3’<br>Rev 5’AATACCAAATGCATGACACCA3’	172 bp	16124	91%	[2]
	Fragment 4	For 5’TCAGCCCATGCCTAACATAA3’<br>Rev 5’TGAGGATGCTCAAGATGCAG3’	143 bp	16252	93%	[2]
	Fragment 5	For 5’CGGAGCATGAATTGTAGCTG3’<br>Rev 5’GTATTGAGGGCGGGATAAAT3’	208 bp	16348	88%	[2]
Modern	LA1	For 5’CAACCTCCTAAAATGAAGACAAG<br>Rev 5’GTATAAGTCTATTGAAAGTTAACAGGA	1451 bp	15280	100%	This study

References

1. Hiendleder S, Lewalski H, Wassmuth R, Janke A. The complete mitochondrial DNA sequence of the domestic sheep (*Ovis aries*) and comparison with the other major ovine haplotype. J Mol Evol. 1998;47(4):441–448.

2. Niemi M, Bläuer A, Iso-Touru T, Nyström V, Harjula J, Taavitsainen JP, et al. Mitochondrial DNA and Y-chromosomal diversity in ancient populations of domestic sheep (*Ovis aries*) in Finland: comparison with contemporary sheep breeds. Genet Sel Evol. 2013;45(2). doi:10.1186/1297-9686-45-2
