# Supplementary material for: Three Thousand Years of Continuity in the Maternal Lineages of Ancient Sheep (Ovis aries) in Estonia
Source: PLoS One. 2016 Oct 12;11(10):e0163676. doi: 10.1371/journal.pone.0163676 (PMC5061334; doi:10.1371/journal.pone.0163676)
Supplement: S3 Table — (PDF) [file pone.0163676.s004.pdf]

S3 Table. Success rate of ancient samples. The success rate of all ancient samples analysed in the current study (n = 134) by (a) region, (b) time period, and (c) conjoined cohorts of region and time period. To examine the possible effect of soil and bedrock conditions on aDNA preservation, comparison was made for the three ancient time periods and geographical regions of northern, western, and southern Estonia, plus the samples from outside Estonia. No significant differences in the preservation of DNA were detected in temporal or spatial cohorts.

	**No of samples**	**Successful with all primer pairs**	**Success rate**	**No of partial samples**	**Total success rate (with all primer pairs + partial)**	**Unsuccessful samples**
**a) Region**						
Northern Estonia	37	29	78%	1	81%	7
Southern Estonia	57	46	81%	5	90%	6
Western Estonia	21	13	62%	2	71%	6
Latvia, Russia, Poland, Greece	19	14	74%	2	84%	3
**Total:**	**134**	**102**	**76%**	**10**	**84%**	**22**
**b) Time period**						
Neolithic / Bronze / Iron Age	55	34	62%	4	69%	17
Iron Age / Middle Ages[1]	2	2	100%	-	100%	-
Middle Ages	54	45	83%	5	93%	4
Early Modern / Modern Period	23	21	91%	1	96%	1
**Total:**	**134**	**102**	**76%**	**10**	**84%**	**22**

[1]Not applicable for temporal analysis.

**c) Success rate (all primer pairs) for every region by time period [2]**			
	Neolithic / Bronze / Iron Age	Middle Ages	Early Modern / Modern Period
Northern Estonia (n = 29)	61%	91%	100%
Southern Estonia (n = 46)	75%	81%	85%
Western Estonia (n = 13)	53%	80%	100%
Latvia, Russia, Poland, Greece (n = 14)	60%	86%	100%

[2]At $p < 0.05$ the differences between preservation of different time and geographical cohorts are not significant ($\chi^2 = 6.0353$, $p = 0.419244$).
