# Supplementary material for: Three Thousand Years of Continuity in the Maternal Lineages of Ancient Sheep (Ovis aries) in Estonia
Source: PLoS One. 2016 Oct 12;11(10):e0163676. doi: 10.1371/journal.pone.0163676 (PMC5061334; doi:10.1371/journal.pone.0163676)
Supplement: S4 Table — (PDF) [file pone.0163676.s005.pdf]

S4 Table. Population pairwise F_{ST} of four temporal cohorts of Estonian ancient and modern Kihnu native sheep. Assessment of genetic differentiation of the Estonian ancient (*n* = 86) and modern (*n* = 44) sheep cohorts using pairwise F_{ST} values shows significant genetic differentiation between Kihnu and medieval, and Kihnu and early modern / modern sheep, as well as between the Bronze/Iron Age and medieval sheep populations. This supports the population expansion discussed in the main text, and the distinctiveness of Kihnu sheep compared to the ancient populations.

	**Bronze/Iron Age**	**Middle Ages**	**Early Modern / Modern Period**	**Kihnu**
**Bronze/Iron Age (*n* = 28)**	0	-	-	-
**Middle Ages (*n* = 39)**	0.03080*	0	-	-
**Early Modern / Modern Period (*n* = 19)**	0.01620	0.01333	0	-
**Kihnu (*n* = 44)**	0.02529	0.13773**	0.09663*	0

* Statistical significance at $0.01 < p < 0.05$.
** Statistical significance at $p < 0.001$.
Statistical significance was estimated using a permutation simulation with 10 000 permutations.
