# Supplementary material for: Three Thousand Years of Continuity in the Maternal Lineages of Ancient Sheep (Ovis aries) in Estonia
Source: PLoS One. 2016 Oct 12;11(10):e0163676. doi: 10.1371/journal.pone.0163676 (PMC5061334; doi:10.1371/journal.pone.0163676)
Supplement: S5 Table — (PDF) [file pone.0163676.s006.pdf]

S5 Table. Haplotype data for ancient and modern samples of this study. Mitochondrial DNA haplotype data for ancient sheep samples (*n* = 102) from Estonia, Latvia, Russia, Poland, and Greece, and modern Kihnu sheep (*n* = 44). Alignment length 559 bp, gaps considered, mapped to GenBank accession NC001941 [1]. A dot indicates a nucleotide similar to that of the reference sequence, a dash indicates an indel, *h* is the haplotype, *n* is the number of samples and *H* is the haplogroup. Among 49 haplotypes six (*H2*, *H29*, *H37*, *H38*, *H45*, *H48*) belong to haplogroup A and rest to haplogroup B.

h	Variable position	*n*	*H*	Individuals
NC001941	1111111111111111111111111111111111111111<br>5555555666666666666666666666666666666666<br>9999999000000000011111122233334444444445<br>7888899012234469902334901444901344456791<br>8234534890262846718034597434311012543373<br>TAAACCCGCATGTTTCAACATCTCCAT-TGGCTCCGTTTG			
H_1	.............C....T......CC..........-..	2	B	4OaJaan1 100OaPadi2
H_2	CG.......GC.CC.TG.T....TT.CC....C..A.-..	1	A	5OaPada1
H_3	..............A...T......CC..........-..	1	B	6OaPost1
H_4	..................T......CC..........-.A	20	B	7OaSoon1 12OaOrdu2 28OaJak1 30OaKea1 37OaSam1 50OaKir2 57OaTou1 58OaTor1 86OaSau1 132OaSau2 LA1 LA2 LA3 LA4 LA5 LA6 LA7 LA8 LA9 LA10
H_5	....T........C....T......CC..........-.A	2	B	8OaVas1 143OaKar7
H_6	..................T......CC..A.......-..	5	B	9OaSpo1 29OaJak2 106OaIlm1 116OaKrak1 129OaAlu2
H_7	........T.........T......CC..........-..	2	B	10OaHuv1 131OaKura1
H_8	..................T......CC..........-..	27	B	11OaLoss1 16OaLoh1 23OaRou1 27OaLut1 45OaOte2 67OaOte3 76OaKar1 82OaSarg1 85OaPai1 87OaNar1 89OaAlu1 91OaTart2 102OaHar1 110OaBot1 111OaLatte1 113OaJaan3 114OaJaan4 119OaRus1 120OaRus2 121OaRus3 122OaPih2 130OaEka3 142OaKar6 LA19 LA24 LA25 LA37
H_9	..................TG.....CC.......A.-..	1	B	13OaRid2
H_10	......T...........T......CC..........-C.	3	B	14OaJaan2 15OaSpo2 19OaTart1
H_11	..................T......CC.AA.......-..	4	B	17OaOte1 62OaPada2 118OaMusu2 141OaJak4
H_12	..................T......CC.......A.-..	5	B	18OaAsva1 21OaPar1 95OaVec1 137OaKar5 138OaKrak3
H_13	.....T.....A......T......CC..........-..	1	B	20OaTal1
H_14	....T.............T......CC.....T.A.-..	1	B	24OaOlu1
H_15	...........A......T......CC......T..-..	1	B	25OaSal1
H_16	.......A..........T......CC..........-..	1	B	31OaKivi2
H_17	..................T..T...CC..........-..	2	B	32OaLoo1 80OaKak1
H_18	....T.............T......CC.A........-..	2	B	34OaPro1 84OaKil1
H_19	..................T...C...CC.........-..	2	B	43OaLih1 140OaJak3
H_20	....T.............T......CC..........-C.	1	B	44OaIru1
H_21	..................T......CC.........C-..	2	B	46OaJoe3 103OaRoos1
H_22	..............C...T......CCC.........-..	1	B	47OaVao3
H_23	..................T......CC.............	2	B	49OaIru2 127OaPar5
H_24	...........A......T......CC..........-..	1	B	51OaVar1
H_25	................T..T......CC........C-..	1	B	52OaSuu2
H_26	............C.....T......CC..A.......-..	1	B	53OaIru3
H_27	.....T..........G.T......CC..........-..	2	B	56OaJoe4 107OaMuuk1
H_28	....T.............T.....GCC..........-C.	1	B	59OaLin1
H_29	CG.......GCACC.TG.T....TT.CC....C..A.-..	2	A	64OaPoi1 105OaAsva3
H_30	..................T......CC......T..-..	11	B	65OaPaa1 128OaPih3 LA13 LA14 LA17 LA26 LA30 LA40 LA41 LA46 LA47
H_31	C.................T......CC..........-..	2	B	69Haa1 115OaVilKv1
H_32	..................T.C....CC..........-..	1	B	77OaVilMu1
H_33	................G.T......CC.AA.......-..	1	B	78OaKures1
H_34	.....T............T......CC..........-..	2	B	79OaEka1 83OaPadi1
H_35	..................TG.....CC..........-..	1	B	81OaPol1
H_36	........T.........T......CC......T..-..	1	B	90OaTer1
H_37	CG.......G.ACC.TGGT....TT.CC....C..A.-..	1	A	93OaKivt1

H_38	CG.......GCACC.TGGT....TT.CC....C..A.-..	1	A	96OaPih1
H_39	...........A.C....T.......CC.........-..	1	B	99OaLohk2
H_40	C.................T.......CC............	1	B	101OaPadi3
H_41	...........A......T.......CC...T.....-..	2	B	109OaMusu1 124OaOlu2
H_42	....T.............T.......CCC........-..	1	B	112OaLatte2
H_43	.G.G...A..........T.......CC.........-..	1	B	123OaSara2
H_44	..................T......GCC....C....-.A	1	B	136OaKar4
H_45	CG.......GCACC.TG.T....TT.CC...TC..A.-..	5	A	LA11 LA15 LA18 LA22 LA29
H_46	.............C....T.......CC........C-..	8	B	LA16 LA20 LA28 LA31 LA32 LA34 LA35 LA42
H_47	..................T.......CCC.....T..-..	1	B	LA23
H_48	CG...T...G.ACC.TG.T....TT.CC....C..A.-..	5	A	LA27 LA39 LA43 LA44 LA45
H_49	..G...............TG......CC.......A.-..	2	B	LA33 LA38

References

1. Hiendleder S, Lewalski H, Wassmuth R, Janke A. The complete mitochondrial DNA sequence of the domestic sheep (*Ovis aries*) and comparison with the other major ovine haplotype. J Mol Evol. 1998;47(4):441–448.
