# Supplementary material for: Three Thousand Years of Continuity in the Maternal Lineages of Ancient Sheep (Ovis aries) in Estonia
Source: PLoS One. 2016 Oct 12;11(10):e0163676. doi: 10.1371/journal.pone.0163676 (PMC5061334; doi:10.1371/journal.pone.0163676)
Supplement: S6 Table — (PDF) [file pone.0163676.s007.pdf]

S6 Table. Haplotype data for ancient and modern samples of this study, and for comparative samples from Finland. Mitochondrial DNA haplotype data for the median-joining network of 523 bp mtDNA D-loop haplotypes presented in Fig 3 (main text): Estonian (*n* = 88), Latvian (*n* = 5), Russian (*n* = 6), Polish (*n* = 2) and Greek (*n* = 1) ancient, and Estonian modern Kihnu sheep (*n* = 44) samples of our study; and Finnish ancient (*n* = 26) and modern (*n* = 32) samples (JX484017–JX484025, JX484035–484057, JX484111–JX484136; [1]). Note that because of the shorter alignment length used in the median-joining networks in Fig 3 and S1 Fig (523 bp compared to the longer alignment of 559 used in the rest of the study), one distinctive mutation recorded in the 559 bp alignment that separated haplotypes *H4* and *H8*, has been merged (see also S5 Table). Therefore, in the current table and in the networks presented in Fig 3 and S1 Fig, the central haplotype in haplogroup B is named *H4*, and *H8* is missing from the list.

h	***n***	***H***	***individuals***
H_1	2	B	4OaJaan1 100OaPadi2
H_2	1	A	5OaPada1
H_3	1	B	6OaPost1
H_4	52	B	7OaSoon1 11OaLoss1 12OaOrdu2 16OaLoh1 23OaRou1 27OaLut1 28OaJak1 30OaKea1 37OaSam1 45OaOte2 50OaKir2 57OaTou1 58OaTor1 67OaOte3 76OaKar1 82OaSarg1 85OaPai1 86OaSau1 87OaNar1 89OaAlu1 91OaTart2 102OaHar1 110OaBot1 111OaLatte1 113OaJaan3 114OaJaan4 119OaRus1 120OaRus2 121OaRus3 122OaPih2 130OaEka3 132OaSau2 142OaKar6 JX484035 JX484042 JX484046 JX484125 JX484130 LA1 LA2 LA3 LA4 LA5 LA6 LA7 LA8 LA9 LA10 LA19 LA24 LA25 LA37
H_5	2	B	8OaVas1 143OaKar7
H_6	9	B	9OaSpo1 29OaJak2 106OaIlm1 116OaKrak1 129OaAlu2 JX484036 JX484037 JX484132 JX484136
H_7	2	B	10OaHuv1 131OaKura1
H_9	1	B	13OaRid2
H_10	3	B	14OaJaan2 15OaSpo2 19OaTart1
H_11	6	B	17OaOte1 62OaPada2 118OaMusu2 141OaJak4 JX484119 JX484134
H_12	8	B	18OaAsva1 21OaPar1 95OaVec1 137OaKar5 138OaKrak3 JX484048 JX484117 JX484131
H_13	5	B	20OaTal1 JX484124 JX484126 JX484127 JX484128
H_14	1	B	24OaOlu1
H_15	1	B	25OaSal1
H_16	4	B	31OaKivi2 JX484039 JX484043 JX484044
H_17	2	B	32OaLoo1 80OaKak1
H_18	2	B	34OaPro1 84OaKil1
H_19	2	B	43OaLih1 140OaJak3
H_20	2	B	44OaIru1 JX484047
H_21	2	B	46OaJoe3 103OaRoos1
H_22	1	B	47OaVao3
H_23	2	B	49OaIru2 127OaPar5
H_24	2	B	51OaVar1 JX484129
H_25	1	B	52OaSuu2
H_26	1	B	53OaIru3
H_27	2	B	56OaJoe4 107OaMuuk1
H_28	1	B	59OaLin1
H_29	3	A	64OaPoi1 105OaAsva3 JX484135
H_30	11	B	65OaPaa1 128OaPih3 LA13 LA14 LA17 LA26 LA30 LA40 LA41 LA46 LA47
H_31	2	B	69Haa1 115OaVilKv1
H_32	1	B	77OaVilMu1
H_33	1	B	78OaKures1
H_34	2	B	79OaEka1 83OaPadi1
H_35	1	B	81OaPol1
H_36	1	B	90OaTer1
H_37	1	A	93OaKivt1
H_38	2	A	96OaPih1 JX484116
H_39	1	B	99OaLohk2
H_40	1	B	101OaPadi3
H_41	3	B	109OaMusu1 124OaOlu2 JX484045
H_42	1	B	112OaLatte2
H_43	1	B	123OaSara2
H_44	1	B	136OaKar4

H_45	5	A	LA11 LA15 LA18 LA22 LA29
H_46	8	B	LA16 LA20 LA28 LA31 LA32 LA34 LA35 LA42
H_47	1	B	LA23
H_48	5	A	LA27 LA39 LA43 LA44 LA45
H_49	2	B	LA33 LA38
H_50	4	B	JX484017 JX484018 JX484024 JX484025
H_51	3	A	JX484019 JX484020 JX484021
H_52	1	B	JX484022
H_53	1	B	JX484023
H_54	1	B	JX484038
H_55	2	B	JX484040 JX484112
H_56	1	B	JX484041
H_57	2	B	JX484049 JX484114
H_58	2	B	JX484050 JX484051
H_59	1	A	JX484052
H_60	4	A	JX484053 JX484054 JX484057 JX484111
H_61	2	B	JX484055 JX484056
H_62	1	B	JX484113
H_63	1	B	JX484115
H_64	1	B	JX484118
H_65	1	B	JX484120
H_66	1	B	JX484121
H_67	1	A	JX484122
H_68	1	B	JX484123
H_69	1	B	JX484133

References

1. Niemi M, Bläuer A, Iso-Touru T, Nyström V, Harjula J, Taavitsainen JP, et al. Mitochondrial DNA and Y-chromosomal diversity in ancient populations of domestic sheep (*Ovis aries*) in Finland: comparison with contemporary sheep breeds. Genet Sel Evol. 2013;45(2). doi:10.1186/1297-9686-45-2
