# Supplementary material for: Three Thousand Years of Continuity in the Maternal Lineages of Ancient Sheep (Ovis aries) in Estonia
Source: PLoS One. 2016 Oct 12;11(10):e0163676. doi: 10.1371/journal.pone.0163676 (PMC5061334; doi:10.1371/journal.pone.0163676)
Supplement: S1 Text — (PDF) [file pone.0163676.s008.pdf]

S1 Text. Sampling and DNA extraction protocol. Bone samples were prepared in a separate room dedicated for sampling archaeological specimens, using separate equipment. Non-disposable equipment (*e.g.* drill bits) was decontaminated between the samples, and latex gloves and protective clothing were worn when handling the specimens. The protocol followed in the study was first described by [1] and amended by [2] with slight modifications.

1. Remove the outer surface of the bone with a drill. Drill 0.5 ml bone powder and divide it into three 2 ml tubes.
2. To dissolve the bone powder, add 900 µl 0.5M EDTA pH 8.0, 100 µl 10M urea and 10 µl Proteinase K (20mg/ml). Incubate overnight in an end-over-end shaker at 55 ºC.
3. Centrifuge 2000 rpm for 5 min. To concentrate the sample, transfer three supernatants in to a single Amicon Ultra-4 30kDa Centrifugal Filter Unit (Merck Millipore, Darmstadt, Germany) and centrifuge 4000 rpm for 8–15 min. The preferred concentrated volume is 200–250 µl (max 300 µl). Move the concentrate into a 2 ml tube.
4. To bind the DNA, add 5 volumes QIAquick (Qiagen, Hilden, Germany) PB-buffer. Vortex and spin down. Load 650 µl of the concentrate onto a QIAquick column and centrifuge 13 000 rpm for 1 min. Discard the flowthrough. Repeat the process until all of the extract has passed through the column.
5. To wash the DNA, add 650 µl QIAquick PE-buffer and centrifuge 13 000 rpm for 1 min. Discard the flowthrough. To dry the column completely, centrifuge 13 000 rpm for 1 min. Discard the flowthrough.
6. To elute the DNA, load 75 µl QIAquick EB-buffer directly onto the column. Incubate 3 min and centrifuge 13 000 rpm for 1 min. Repeat the process. The total volume of the DNA extract is 150 µl.

References

1. Yang DY, Eng B, Waye JS, Dudar JC, Saunders SR. Technical note: improved DNA extraction from ancient bones using silica-based spin columns. Am J Phys Anthropol. 1998;105(4):539–543. doi:10.1002/(SICI)1096-8644(199804)105:4<539::AID-AJPA10>3.0.CO;2-1
2. Niemi M, Bläuer A, Iso-Touru T, Nyström V, Harjula J, Taavitsainen JP, et al. Mitochondrial DNA and Y-chromosomal diversity in ancient populations of domestic sheep (*Ovis aries*) in Finland: comparison with contemporary sheep breeds. Genet Sel Evol. 2013;45(2). doi:10.1186/1297-9686-45-2
