# Supplementary material for: Three Thousand Years of Continuity in the Maternal Lineages of Ancient Sheep (Ovis aries) in Estonia
Source: PLoS One. 2016 Oct 12;11(10):e0163676. doi: 10.1371/journal.pone.0163676 (PMC5061334; doi:10.1371/journal.pone.0163676)
Supplement: S2 Text — (PDF) [file pone.0163676.s009.pdf]

S2 Text. Radiocarbon dating. The results in this study rely to a certain extent on the accurate dating of the archaeological sheep remains. The majority of samples were dated based on archaeological context, that is, through the associated finds and site stratigraphy. As the bones were recovered from a variety of sites (settlements, hillforts, cemeteries) and excavated at different times and under different conditions, the documentation and therefore the precision of contextual dating could vary. Nevertheless, care was taken to sample the bones with an applicable context. For most of the samples with unclear context radiocarbon dating was conducted (S1 Table).

Fourteen samples were radiocarbon dated by AMS in the Poznań Radiocarbon Laboratory (all calibrations according to IntCal13 atmospheric curve [1]; OxCal v4.2.3 [2]; r:5) and SUERC Radiocarbon Dating Laboratory (all calibrations according to IntCal13 atmospheric curve [1]; OxCal v.4.1.7 [3]; r:5). For three samples the datings have previously been reported in [4].

Some of the dating results were somewhat surprising, as seven samples produced dates significantly later than those based on the archaeological context: five of those come from Middle Bronze and Early Iron Age stone graves, which have complicated contextual data as well as poor documentation; and other two derive from a settlement site and field remains, which also can yield chronologically mixed archaeological material. These results raise awareness of the complexity of archaeological contexts and should be noted when working on and interpreting the past faunal remains.

References

1. Reimer PJ, Bard E, Bayliss A, Beck JW, Blackwell PG, Bronk Ramsey C, et al. IntCal13 and Marine13 Radiocarbon Age Calibration Curves 0–50,000 Years cal BP. Radiocarbon. 2013;55*(*4):1869–1887. doi:10.2458/azu_js_rc.55.16947
2. Bronk Ramsey C, Lee S. Recent and Planned Developments of the Program OxCal. Radiocarbon. 2013;55(2-3):720–730. doi:10.2458/azu_js_rc.55.16215
3. Bronk Ramsey C, Dee M, Lee S, Nakagawa T, Staff R. Developments in the calibration and modelling of radiocarbon dates. Radiocarbon. 2010;52(3):953–961.
4. Rannamäe E, Lõugas L, Niemi M, Kantanen J, Maldre L, Kadõrova N, et al. Maternal and paternal genetic diversity of ancient sheep in Estonia from the Bronze Age to the Post-Medieval Period, and comparison with other regions in Eurasia. Anim Genet. 2016;47(2):208–218. doi:10.1111/age.12407
